# Tackling FGFR3-driven bladder cancer with a promising synergistic FGFR/HDAC targeted therapy

**DOI:** 10.1038/s41698-023-00417-5

**Published:** 2023-07-21

**Authors:** Zechen Wang, Viswanathan Muthusamy, Daniel P. Petrylak, Karen S. Anderson

**Affiliations:** 1 Department of Pharmacology, Yale University School of Medicine, 333 Cedar St., New Haven, CT 06520 USA; 2 Center for Precision Cancer Modeling, Yale School of Medicine, New Haven, CT USA; 3 Smilow Cancer Center, Yale University, New Haven, CT USA; 4 Department of Molecular Biophysics and Biochemistry, Yale University School of Medicine, 333 Cedar St., New Haven, CT 06520 USA

**Keywords:** Bladder cancer, Targeted therapies, Oncogenes

## Abstract

Bladder cancer (BC) is one of the most prevalent malignancies worldwide and FGFR3 alterations are particularly common in BC. Despite approval of erdafitinib, durable responses for FGFR inhibitors are still uncommon and most patients relapse to metastatic disease. Given the necessity to discover more efficient therapies for BC, herein, we sought to explore promising synergistic combinations for BC with FGFR3 fusions. Our studies confirmed the synergy between FGFR and HDAC inhibitors in vitro and demonstrated its benefits in vivo. Mechanistic studies revealed that quisinostat can downregulate FGFR3 expression by suppressing FGFR3 translation. Additionally, quisinostat can also sensitize BC cells to erdafitinib by downregulating HDGF. Furthermore, the synergy was also confirmed in BC cells with FGFR3 S249C. This study discovers a new avenue for treatment of FGFR3-driven BC and uncovers new mechanistic insights. These preclinical studies pave the way for a direct translation of this combination to early phase clinical trials.

## Introduction

Bladder cancer (BC) is the 10th most frequent malignancy and 13th most common cause of death worldwide^1^, accountable for more than 570,000 new cases and over 200,000 deaths in 2020^2,3^. The incidence of BC in men is approximately four times higher than in women, making BC the 4th most common cancer in men^4^. The majority of BC (~70–80%) are low-grade, non-muscle invasive bladder cancer (NMIBC) upon diagnosis, and ~20% of new cases are high-grade, muscle invasive bladder cancer (MIBC)^5^. Traditionally, BC patients have been treated by surgical and pharmacological treatments, or the combination of both, depending on the tumor status^6^. However, 70–80% of NMIBC patients will develop at least one recurrence within 5 years after the initial treatment, and 10–20% of these patients will progress into MIBC^7^. In addition, the recurrence and metastasis rates for MIBC patients are around 50%, with the 5-year overall survival less than 50%^8^. In spite of the recent development of new therapeutic strategies, durable responses are still uncommon and the majority of patients relapse and succumb to metastatic disease^1^. Therefore, the discovery of new therapeutic modalities is still urgently needed for BC patients.

Fibroblast growth factor receptor (FGFR) belongs to receptor tyrosine kinase (RTK) superfamily^9^. There are four family members of FGFR (FGFR1–4) and the proper regulation of FGFR signaling is essential for a number of cellular processes, such as cell proliferation, migration, and survival^10–12^. FGFR3 alterations, including FGFR3 activating mutations, FGFR3 fusions, and FGFR3 overexpression, are especially common in BC^13^. In particular, FGFR3 mutations and fusions have been detected in up to 80% of NMIBC patients and ~10–20% of MIBC patients^14^. In addition, FGFR3 overexpression has also been reported in ~40–50% of MIBC patients^15^, making FGFR3 a promising target for BC treatment. In 2019, FDA approved erdafitinib, a selective FGFR inhibitor (FGFRi), for the treatment of locally advanced or metastatic bladder cancer with FGFR2 or FGFR3 alterations^16^. However, unfortunately, despite an objective response rate of 40%, complete responses were rare (3%) and the median duration of response is 5.6 months^5,17,18^. In addition, interestingly, one clinical trial of erdafitinib suggested that BC patients with FGFR fusions seems to have a lower response rate than patients with FGFR3 activating mutations (16% vs. 49%)^16^. Thus, the development of new treatment options for BC patients with FGFR aberrations are still highly desired, especially for patients with FGFR fusions.

Histone deacetylases (HDACs) are a family of epigenetic regulators, responsible for the removal of acetyl group from lysine residues in histone^8^. HDACs contain 18 family members which can be categorized into four classes^8^. Overexpression of Class I HDACs (HDAC1/2/3/8) is known to be related with multiple cancers, including ovarian, gastric, and prostate cancers^19–21^. Interestingly, upregulation of Class I HDACs in BC has been reported in a number of studies^22–26^, making HDAC inhibitors (HDACi) an important field in drug development for BC treatment. However, despite the prevalence of HDAC upregulation in bladder cancer, it seems like the effects of HDACi as monotherapy for BC patients are quite limited in clinical trials^27^.

Our previous studies developed novel models of cholangiocarcinoma with FGFR2 fusions to discover more efficient therapeutics^28^. This included unbiased high-throughput screening that identified pemigatinib, another selective FGFR inhibitor, and quisinostat, a second-generation pan-HDACi, as a synergistic combination. Therefore, due to the high demand for the discovery of more potent therapeutic options for BC patients and the necessity to further improve targeted therapies, in this study, we sought to further explore the combination between erdafitinib and quisinostat in BC with FGFR3 aberrations and understand the underlying mechanisms. We first confirmed the synergy between erdafitinib and quisinostat in three BC cell lines (SW780, RT112, and RT4 cells) with FGFR3 fusions in vitro. We then established xenografts from the FGFR3 fusion-positive BC cells and further validated that the combinational treatment can significantly enhance the inhibition of tumor growth and prolong the survival in vivo. In order to dissect the molecular mechanisms behind the synergy, we revealed that quisinostat can downregulate FGFR3 expression by inhibiting FGFR3 protein translation. In addition, quisinostat can also sensitize BC cells to erdafitinib by downregulating hepatoma-derived growth factor (HDGF). To further extend the application of this combination, we verified that erdafitinib and quisinostat are also synergistic in BC cells with an FGFR3 S249C mutation, which is the most prevalent FGFR3 activating mutations in BC^14^.

Our study discovers a new avenue for treatment of BC patients with FGFR3 aberrations. And we provide innovative mechanistic insights behind the synergy of FGFR and HDAC inhibitors, which may guide and contribute to future development of new therapeutics as well as undercovering new prognostic biomarkers. Our results present the preclinical proof of principle that is necessary for the design of an early phase clinical trial involving the application of the combination of FGFR inhibitors with HDAC inhibitors on BC patients with FGFR aberrations.

## Results

### Erdafitinib and quisinostat are synergistic in FGFR3 fusion-positive BC cells

**Fig. 1:**
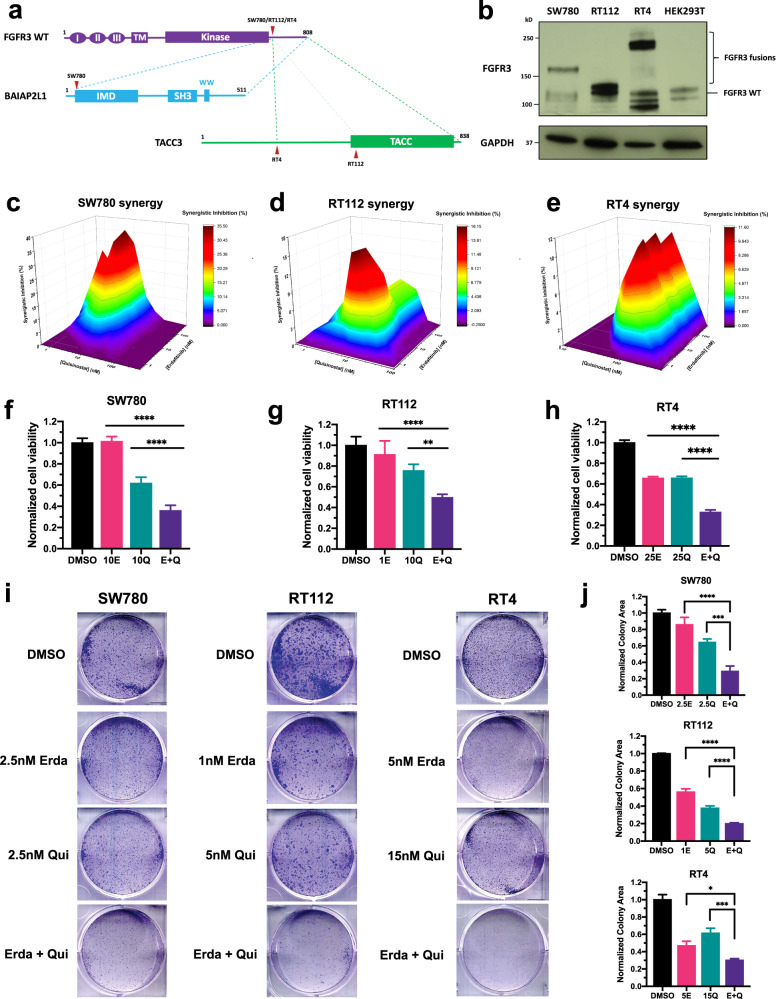
Erdafitinib and quisinostat are synergistic in FGFR3 fusion-positive BC cells. **a** Schematic diagram of FGFR3 fusions in SW780, RT112, and RT4 cells. All three FGFR3 fusions share a common construct, where the intact extracellular region, transmembrane helix, and intracellular kinase domain of FGFR3 are well-maintained, with only the C terminal tail of FGFR3 replaced by a fusion partner. Red arrowheads represent the breakpoints of FGFR3 fusions in each cell line. FGFR3-BAIBP2L1 fusion is indicated by blue dashed line. FGFR3-TACC3 fusion in RT4 cells is indicated by green dashed line. The alternative breakpoint in TACC3 for FGFR3-TACC3 fusion in RT112 cells is indicated by light green dashed line. I/II/III, immunoglobulin-like domain (Ig) I/II/III; TM, transmembrane domain; IMD, IRSp53/MIM homology domain; TACC, transforming acidic coiled-coil domain. **b** Western blots showing FGFR3 fusions in SW780 (FGFR3-BAIBP2L1), RT112 (FGFR3-TACC3), and RT4 (FGFR3-TACC3) cells. HEK293T cells were used as FGFR3 WT control. GAPDH was used as standard loading control. Positions for FGFR3 fusions and FGFR3 WT are indicated on the right. **c**–**e** MacSynergy II calculation (95% confidence interval) of the synergy between erdafitinib and quisinostat in SW780 cells (**c**), RT112 cells (**d**), and RT4 cells (**e**). Cells were treated by different concentrations of erdafitinib and/or quisinostat for 3 days. And cell viabilities were determined by WST-1 assay and normalized to DMSO control. Synergistic inhibition was calculated by MacSynergy II. Synergistic inhibition above 0 means synergy, equal to 0 indicates additivity, and below 0 suggests antagonism. **f**–**h** Cell viability of SW780 cells (**f**), RT112 cells (**g**), and RT4 cells (**h**) by the treatment of erdafitinib and/or quisinostat. Cells were treated by erdafitinib and/or quisinostat for 3 days. And cell viabilities were determined by WST-1 assay and normalized to DMSO control. Data were plotted as mean ± standard deviation from three biological replicates and statistics were calculated by one-way ANOVA (**p < 0.01; ****p < 0.0001). **i** Clonogenic assays of SW780, RT112, and RT4 cells, under the treatment of erdafitinib, quisinostat, or the combination. Cells were seeded in 6-well plates with DMSO control, erdafitinib, quisisnostat, or the combination and cultured for 9 days. **j** Quantification of (**i**). Colony area was quantified by ImageJ and normalized to DMSO control. Data were plotted as mean ± standard deviation from three biological replicates and statistics were calculated by one-way ANOVA (*p < 0.05; ***p < 0.001; ****p < 0.0001).

In order to confirm the synergy between erdafitinib and quisinostat, we used three BC cell lines with FGFR3 fusions: SW780 cells with FGFR3-BAIBP2L1 fusion, RT112 cells with FGFR3-TACC3 fusion, and RT4 cells with FGFR3-TACC3 fusion. All three FGFR3 fusions share a common construct, where the extracellular region, transmembrane helix, and intracellular kinase domain of FGFR3 are well-maintained, with only FGFR3 C terminal tail replaced by a fusion partner (Fig. 1a). These FGFR3 fusions have been reported to cause constitutive activation of FGFR signaling and therefore, are usually regarded as driver mutations for tumorigenesis^29^. The existence of FGFR3 fusions was validated by western blot (Fig. 1b). All three BC cell lines contain one FGFR3 species with a higher molecular weight than FGFR3 WT (Fig. 1b), which is in consistence with previous reports^30,31^. Of note, since RT112 and RT4 cells have distinct break points of FGFR3-TACC3 fusions (Fig. 1a)^31^, the molecular weights of FGFR3-TACC3 fusions are different in these two cells (Fig. 1b). We also examined HDAC expression level in all BC cell lines. Compared to TRT-HU-1 cells, an immortalized healthy bladder epithelial cells, HDAC1/2/3 are all overexpressed in bladder cancer cells (Supplementary Fig. 1).

To determine possible synergy between erdafitinib and quisinostat in FGFR3 fusion-positive BC, we treated all three BC cells with erdafitinib and/or quisinostat for 3 days and then examined cell viability by WST-1 assay. Synergy was then assessed by the program MacSynergy II^32^. The two drugs are synergistic if the synergistic inhibition is >0, additive if =0, and antagonistic if <0. As shown in Fig. 1c–e, for all three BC cells, the synergistic inhibition is >0 for most of the concentration pairs we tested at low nanomolar concentrations, suggesting the synergy of erdafitinib and quisinostat in BC with FGFR3 fusions. In addition, compared to each individual drug treatment, the combinational treatment can further decrease cell viabilities in all three BC cells (Fig. 1f–h). Furthermore, combining erdafitinib with quisinostat can decrease the IC_50_ of quisinostat by 45–70% in all three BC cells (Table 1), which further indicates the synergy between erdafitinib and quisinostat.

**Table 1:** Effects of erdafitinib on quisinostat IC_50_.

Cell lines	Quisinostat IC_50_ (nM)^a^		Fold change
	DMSO	Erdafitinib	
SW780	26 ± 14	13 ± 8^b^	0.50
RT112	27 ± 16	8 ± 4^b^	0.30
RT4	44 ± 18	24 ± 5^b^	0.55
UM-UC-14	23 ± 4	9 ± 2^c^	0.39

^a^Data represented by mean ± standard deviation from three biological replicates.

^b^Quisinostat IC_50_ under 50 nM erdafitinib.

^c^Quisinostat IC_50_ under 5 nM erdafitinib.

The synergy was also further verified by clonogenic assays. Compared to the treatment by each drug separately, the combinational treatment can achieve much more significant inhibition on clonogenic growth in all three BC cell lines (Fig. 1i, j). Therefore, our data demonstrates that erdafitinib and quisinostat are synergistic in BC with FGFR3 fusions in vitro.

We also examined the combinational effects of erdafitnib and quisinostat in an immortalized healthy human bladder epithelial cell line, TRT-HU-1. Erdafitinib and quisinostat only have additive effects in TRT-HU-1 cells (Supplementary Fig. 2a). Furthermore, erdafitinib doesn’t significantly affect cell viability, even though quisinostat exhibits cytotoxicity effects at higher concentrations to some extent (Supplementary Fig. 2b), which is very much in line with the toxicity profile of HDAC inhibitors in other studies and clinical trials^27,33,34^. More importantly, combining erdafitinib with quisinostat does not potentiate the cytotoxicity of quisinostat in TRT-HU-1 cells (Supplementary Fig. 2b). These data further strengthen the merits of our combinational treatment, which allows use of a lower dose of quisinostat to reduce its toxicity without sacrificing its anti-tumor potency.

### Combinational treatment by erdafitinib and quisinostat can enhance anti-tumor effects and prolong survival of BC with FGFR3 fusions in vivo

**Fig. 2:**
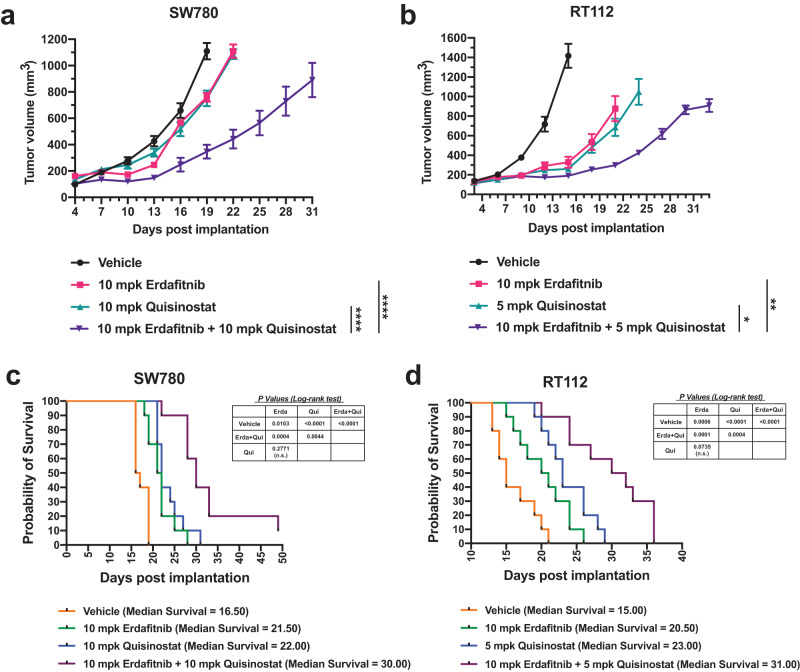
Combinational treatment by erdafitinib and quisinostat can enhance anti-tumor effects and prolong survival of BC with FGFR3 fusions in vivo. **a**, **b** Tumor volumes of SW780 xenografts (**a**) under the treatment of vehicle, 10 mg/kg erdafitinib, 10 mg/kg quisinostat, or the combination. And tumor volumes of RT112 xenografts (**b**) under the treatment of vehicle, 10 mg/kg erdafitinib, 5 mg/kg quisinostat, or the combination. Data were plotted as mean ± SEM (*n* = 5). Statistics were calculated by one-way ANOVA based on the tumor volume at Day 21 for SW780 xenografts and Day 22 for RT112 xenograft (*p < 0.05; **p < 0.01; ****p < 0.0001). **c**, **d** Overall survival of SW780 xenografts (**c**) under the treatment of vehicle, 10 mg/kg erdafitinib, 10 mg/kg quisinostat, or the combination (*n* = 10). And overall survival of RT112 xenografts (**d**) under the treatment of vehicle, 10 mg/kg erdafitinib, 5 mg/kg quisinostat, or the combination (*n* = 10). Log-Rank (Mantel-Cox) test was used to test for significance, as indicated in the figure. n.s., non-significant.

We next explored the combinational effects between erdafitinib and quisinostat in SW780 and RT112 xenografts in vivo. Xenografts were established by injecting the cells subcutaneously into the right flank of immune deficient Rag2/IL2RG double knockout mice. When tumors reached a palpable and similar size, erdafitinib, quisinostat, or the combination were administrated. Similar to in vitro results, compared to the treatment by each drug separately, co-treatment by erdafitinib and quisinostat can enhance the inhibition of tumor growth in both xenografts (Fig. 2a, b). Furthermore, the combinational treatment can also significantly prolong the survival of mice (Fig. 2c, d). The median survival for the combination treatment was prolonged to 30 days in SW780 xenografts, compared to 21.5 and 22 days with erdafitinib and quisinostat treatment, respectively (Fig. 2c). Likewise, the median survival was further extended to 31 days by the combinational treatment in RT112 xenografts, compared to 20.5 and 23 days when treated by erdafitinib and quisinostat separately (Fig. 2d). Of note, in order to avoid potential toxicity, the dose administration was tapered down to once every 3 days after about 2 weeks of daily dosing. Therefore, the dosages of erdafitinib and quisinostat in our study were much lower than the dosages used in previous reports^35,36^. With this treatment regimen at much lower dosages, no apparent toxicity has been noticed per IACUC mandated visual body condition observations. These data further highlight the benefits of our combinational treatment in vivo and indicate that we can achieve sufficient anti-tumor potency with reduced toxicity by using much lower dosages of both drugs.

### Quisinostat treatment can downregulate the expression level of FGFR3

**Fig. 3:**
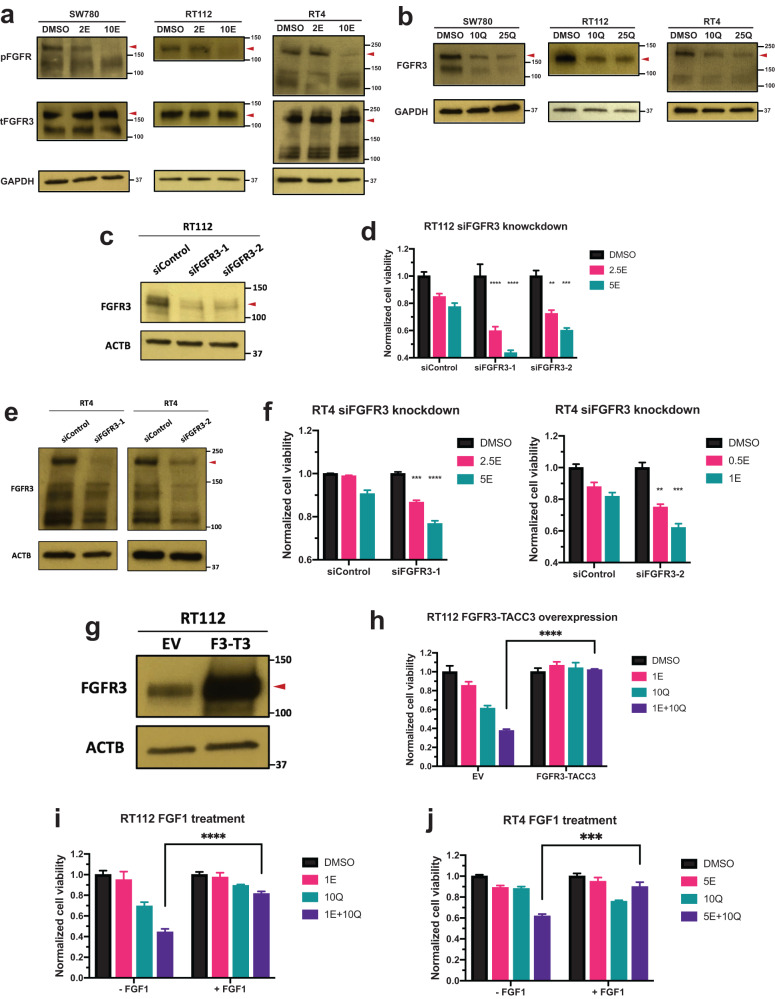
Quisinostat treatment can downregulate the expression level of FGFR3. **a** Western blots showing the phospho-FGFR (pFGFR) level treated by erdafitinib in SW780, RT112, and RT4 cells. Cells were first treated by erdafitinib or DMSO in no serum media for 3 h. FGFR signaling was then stimulated by adding 50 ng/ml FGF1 and 10 ug/ml heparin and examined by western blotting. GAPDH was used as standard loading control. Red arrowheads represent the position of FGFR3 fusions. 2/10E, 2/10 nM erdafitinib. **b** Western blots showing the FGFR3 expression level treated by quisinostat in SW780, RT112, and RT4 cells. Cells were first treated by quisinostat or DMSO for 2 days and then harvested for western blotting. GAPDH was used as standard loading control. Red arrowheads represent the position of FGFR3 fusions. 10/25Q, 10/25 nM erdafitinib. **c**, **e** Western blots showing the FGFR3 expression level after siRNA knockdown by both siRNAs of FGFR3 (siFGFR3-1 and -2) in RT112 cells (**c**) and RT4 cells (**e**). β-actin (ACTB) was used as standard loading control. Red arrowheads represent the position of FGFR3 fusions. **d**, **f** Cell viabilities of RT112 cells (**d**) and RT4 cells (**f**) treated by erdafitinib with or without FGFR3 knockdown. Cells were treated by erdafitinib for 3 days. And cell viabilities were determined by WST-1 assay and normalized to DMSO control for each cell line. Data were plotted as mean ± standard deviation from three biological replicates and statistics were calculated by one-way ANOVA (**p < 0.01; ***p < 0.001; ****p < 0.0001). siControl, siRNA of non-targeting control; 0.5/1/2.5/5E, 0.5/1/2.5/5 nM erdafitinib. **g** Western blots showing the FGFR3-TACC3 overexpression in RT112 cells. β-actin (ACTB) was used as standard loading control. Red arrowhead represents the position of FGFR3 fusion. EV, empty vector; F3-T3, FGFR3-TACC3. **h** Cell viabilities of RT112 cells treated by erdafitinib and/or quisinostat with or without FGFR3-TACC3 overexpression. Cells were treated by erdafitinib and/or quisinostat for 3 days. And cell viabilities were determined by WST-1 assay and normalized to DMSO control for each cell line. Data were plotted as mean ± standard deviation from three biological replicates and statistics were calculated by t tests (****p < 0.0001). EV, empty vector; 1E, 1 nM erdafitinib; 10Q, 10 nM quisinostat. **i**, **j** Cell viabilities of RT112 cells (**i**) and RT4 cells (**j**) treated by erdafitinib and/or quisinostat with or without adding FGF1. Cells were treated by erdafitinib and/or quisinostat for 3 days with or without adding 50 ng/ml FGF1 and 10 ug/ml heparin. And cell viabilities were determined by WST-1 assay and normalized to DMSO control for each condition. Data were plotted as mean ± standard deviation from three biological replicates and statistics were calculated by t tests (***p < 0.001; ****p < 0.0001). 1/5E, 1/5 nM erdafitinib; 5/10Q, 5/10 nM quisinostat.

Next, we intended to dissect the underlying mechanisms behind the synergy of erdafitinib and quisinostat. We first examined the sensitivity of FGFR signaling to erdafitinib. The activation of FGFR3 activity, reflected by phospho-FGFR, can be significantly inhibited by low nanomolar erdafitinib in all three BC cells (Fig. 3a). Then we investigated the effects of quisinostat on FGFR signaling. Interestingly, quisinostat can downregulate the expression of FGFR3 in all three BC cells at low nanomolar concentrations (Fig. 3b and Supplementary Fig. 3). We also examined the effects of quisinostat on EGFR/ErbB family members. Quisinostat treatment does not affect EGFR expression (Supplementary Fig. 4a). In addition, even though ErbB2–4 are downregulated by quisinostat in SW780 cells (Supplementary Fig. 4b), ErbB2 and ErbB3 are not affected by quisinostat in RT4 and RT112 cells (Supplementary Fig. 4b). ErbB4 is even upregulated by quisinostat in RT4 and RT112 cells (Supplementary Fig. 4b). These data further confirm the specificity of quisinostat on FGFR3 downregulation. Therefore, one of the possible mechanisms of the synergy is that quisinostat can sensitize the cells to erdafitinib by downregulating FGFR3.

In order to further corroborate this hypothesis, we knocked down FGFR3 by siRNA in RT112 and RT4 cells, and then examined the sensitivity of the cells to erdaftinib. We hypothesized that if FGFR3 downregulation by quisinostat can mediate the synergy of the two drugs, knocking down FGFR3 by siRNA should also allow erdifitinib to achieve stronger inhibitory effects. Of note, for experimental design, we wanted to achieve only partial FGFR3 knockdown (Fig. 3c, e), so that there is still residual FGFR3 expression level for erdafitinib to act upon. FGFR3 knockdown can decrease cell viabilities in both cells (Supplementary Fig. 5). Importantly, partial FGFR3 knockdown can make erdafitinib achieve more potent inhibition in both RT112 and RT4 cells (Fig. 3d, f). In addition, we also overexpressed FGFR3-TACC3 in RT112 cells (Fig. 3g), and FGFR3-TACC3 overexpression can significantly rescue RT112 cells from the combinational treatment (Fig. 3h). Similarly, we also treated RT112 and RT4 cells by the drug combination with or without the supplement of FGF1 ligand. As demonstrated in Fig. 3i, j, adding FGF1 can significantly reduce the inhibitory effects of the combinational treatment. Noticeably, neither overexpressing FGFR3-TACC3 or adding FGF1 can significantly enhance cell proliferation (Supplementary Fig. 6). This is possibly because RT112 and RT4 cells are already well-transformed cells with FGFR3 overexpressed and hyperactivated. Overall, our results indicate that FGFR3 downregulation by quisinostat might be one of the possible mechanisms behind the synergy.

**Fig. 4:**
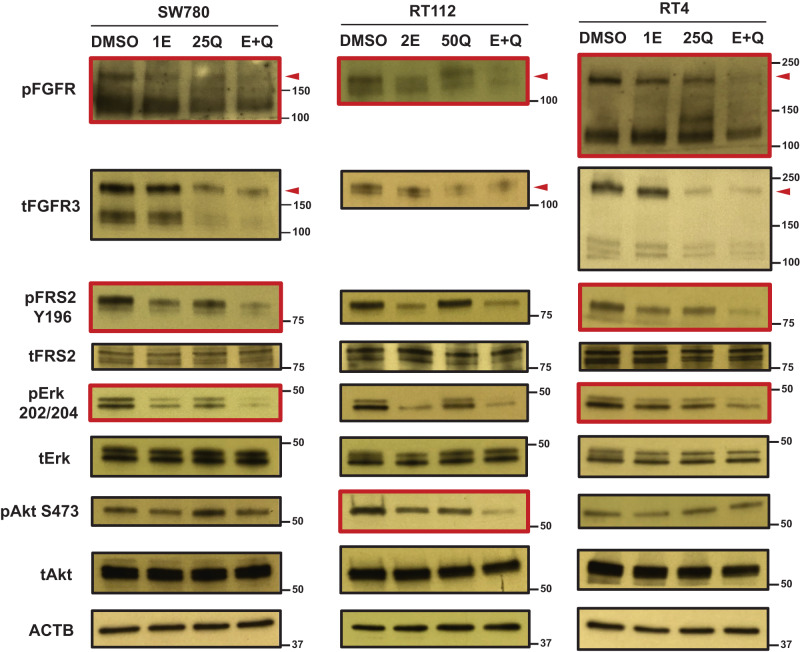
Quisinostat can concomitantly inhibit FGFR signaling with erdafitinib. Western blots showing the combinational effects of erdafitinib and quisinostat on FGFR signaling activation. Cells were first treated by quisinostat or DMSO control for 2 days, followed by the treatment by erdafitinib or DMSO control in no serum media for 3 h. Then FGFR signaling was stimulated by 50 ng/ml FGF1 + 10 µg/ml heparin and analyzed by western blotting. β-actin (ACTB) was used as standard loading control. Red arrowheads represent the position of FGFR3 fusions. Red boxes represent the signaling pathways that can be further inhibited by the combinational treatment, compared to each individual drug treatment alone. 1E, 1 nM erdafitinib; 2E, 2 nM erdafitinib; 25Q, 25 nM quisinostat; 50Q, 50 nM quisinostat; E + Q, the combination of erdafitinib and quisinostat.

We also further explored the effects of this combination on FGFR signaling. We first treated all three BC cells by quisinostat for 2 days, followed by 3 h erdafitinib treatment in no serum starvation media. FGFR signaling was then activated by FGF1 and examined by western blotting. As shown in Fig. 4, phospho-FGFR can be further inhibited by the combination in all three BC cells, compared to each individual inhibitor. Intriguingly, when we looked into FGFR downstream signaling, for SW780 and RT4 cells, the combination can achieve synergistic inhibition on FRS2 and Erk signaling, whereas Akt signaling activation cannot be affected by either of the inhibitors nor the combination (Fig. 4). On the contrary, for RT112 cells, Akt signaling not only can be inhibited by each inhibitor separately, co-treatment by erdafitinib and quisinostat can further suppress Akt signaling activation (Fig. 4). However, only erdafitinib can inhibit Erk signaling in RT112 cells and the combinational treatment failed to achieve further inhibition. We also extended the erdafitinib treatment to 1 day and we observed similar results (Supplementary Fig. 7). While additional mechanisms behind this difference are currently under investigation, overall, our data demonstrates that the combination of quisinostat and erdafitinib can achieve stronger inhibition on FGFR signaling, compared to erdafitinib alone.

### Quisinostat downregulates FGFR3 expression by suppressing FGFR3 translation

**Fig. 5:**
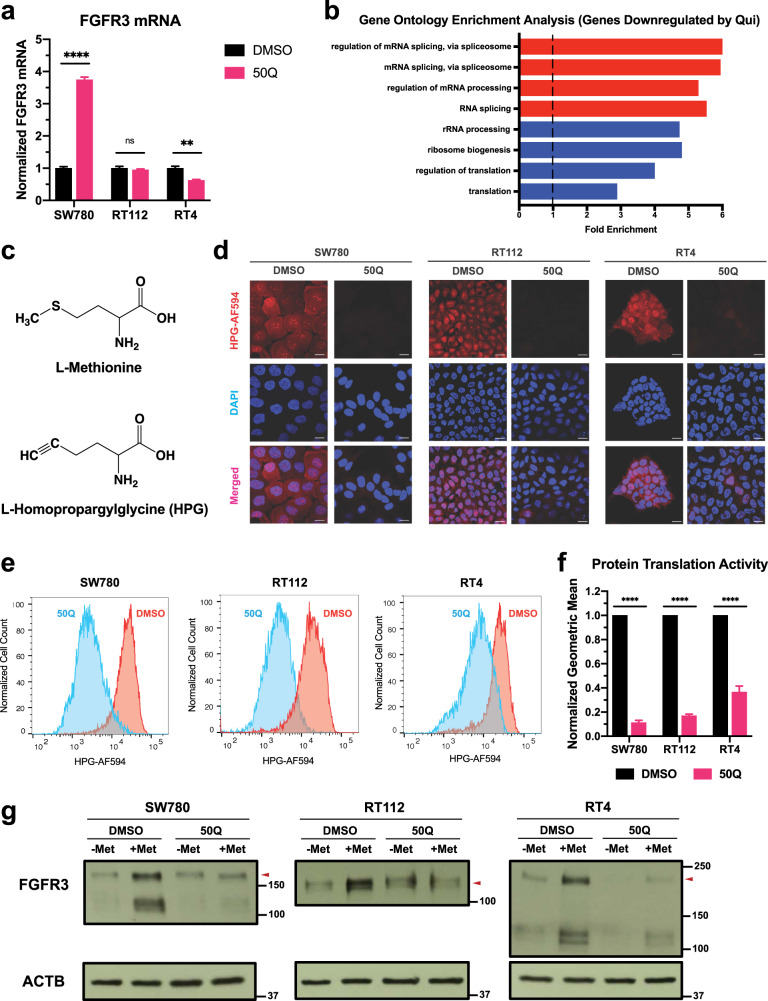
Quisinostat downregulates FGFR3 fusions by suppressing FGFR3 translation. **a** RT-qPCR results showing the FGFR3 mRNA level with or without quisinostat treatment in all three BC cells. All results were first normalized to β-actin (ACTB) loading control and then normalized to DMSO control of each cell line. Data were plotted as mean ± SEM from four biological replicates and statistics were calculated by t tests (ns, non-significant; **p < 0.01; ****p < 0.0001). **b** Gene ontology enrichment analysis of the RNA-seq results reveals that genes involved in protein translation are more likely to be downregulated by quisinostat in all three BC cells (log_2_Fold-change < −0.3). **c** Chemical structures of L-methionine and L-homopropargylglycine (HPG). **d** Microscopic results of total protein translation assays with or without the treatment of quisinostat. Cells were first treated by quisisnostat for 2 days and then analyzed by total protein translational assays. Scale bar: 16 µm. **e** Flow cytometry results of total protein translation assays with or without the treatment of quisinostat. Cells were first treated by quisisnostat for 2 days and then analyzed by total protein translational assays. Cell counts were normalized to the mode of each sample. **f** Normalized geometric means of (**e**). Quisinostat treatment can decrease total protein translation to ~10–40%, depending on the cell line. Geometric means of each sample were normalized to the DMSO control of each cell line. Data were plotted as mean ± standard deviation from three biological replicates and statistics were calculated by t tests (****p < 0.0001). **g** Western blots showing FGFR3 translation with or without quisinostat treatment in all three BC cells. Cells were first treated by quisinostat or DMSO for 2 days and then starved by no L-methionine media overnight. L-methionine was then added into the culture media and cells were incubated for another 8 h. After that, cells were harvested for western blotting. β-actin (ACTB) was used as standard loading control. 50Q, 50 nM quisinostat; Met, L-methionine.

We demonstrated the downregulation of FGFR3 expression by quisinostat (Fig. 3b). However, RT-qPCR results using the primers recognizing the N-terminus of FGFR3 revealed that FGFR3 mRNA level cannot be downregulated by quisinostat in all three BC cells (Fig. 5a). Especially for SW780 cells, quisinostat can even enhance the FGFR3 mRNA level (Fig. 5a). Therefore, we hypothesized that instead of transcription, some other biological processes can also be affected by quisinostat. We then performed RNA-seq analyses on all three BC cell lines with or without quisinostat treatment. For those genes that were downregulated by quisinostat in all three BC cells (log_2_Fold-change < −0.3), we performed gene ontology enrichment analysis and revealed that those genes involved in protein translation are more likely to be inhibited by quisinostat (Fig. 5b). Thus, it is plausible that quisinostat can downregulate FGFR3 expression by inhibiting FGFR3 translation.

In order to further substantiate this hypothesis, we examined the total protein translation activities with or without quisinostat treatment. We first treated BC cells by 50 nM quisinostat for 2 days, and then we substituted L-methionine in the culture media with L-homopropargylglycine (HPG) (Fig. 5c). HPG is a L-methionine derivative that can be incorporated into newly synthesized nascent peptides in place of L-methionine. After incorporation, through a click chemistry reaction with Alexa Fluor 594 (AF594), HPG allows visualization using fluorescence. Therefore, total protein translation activities in cells can be reflected by the fluorescent signal of AF594 detected by microscopy (Fig. 5d) or flow cytometry (Fig. 5e, f). As shown in Fig. 5d by confocal microscopy, quisinostat can significantly inhibit the total protein translation in all three BC cell lines. Total protein translation activities were also analyzed and quantified by the geometric mean of each sample from flow cytometry (Fig. 5e, f). Quisinostat can inhibit the total protein translation activities to ~10–40%, compared to DMSO control, in all three BC cells (Fig. 5f).

We next explored the effects of quisinostat on FGFR3 protein translation. After treatment with 50 nM quisinostat for 2 days, cells were starved by no L-methionine media overnight to halt the protein translation. Then L-methionine was added into the media and cells were incubated for another 8 h to re-start the translation. FGFR3 expression level was then determined by western blotting. For all three BC cells, adding back L-methionine can increase the expression level of FGFR3 for the DMSO control group (Fig. 5g and Supplementary Fig. 8), suggesting that FGFR3 translation was successfully resumed. On the other hand, FGFR3 expression level is not increased at all or only to a much lesser extent for the quisinostat treated group (Fig. 5g and Supplementary Fig. 8), which indicates that FGFR3 translation is inhibited by quisinostat. In summary, our data confirms that quisinostat may downregulate FGFR3 expression level by suppressing FGFR3 translation.

### Quisinostat can also sensitize BC cells to erdafitinib by downregulating HDGF

**Fig. 6:**
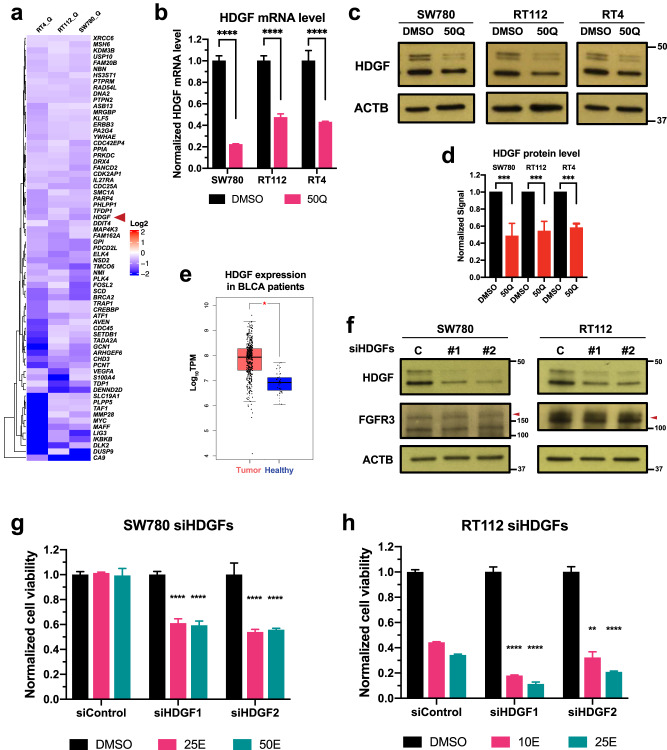
Quisinostat can also sensitize BC cells to erdafitinib by downregulating HDGF. **a** RNA-seq results showing the representative genes that are downregulated by quisinostat in all three BC cells. **b** RT-qPCR results showing the HDGF mRNA level with or without quisinostat treatment. Cells were treated by quisisnostat for 2 days and then harvested for RT-qPCR. All results were first normalized to β-actin (ACTB) loading control and then normalized to DMSO control of each cell line. Data were plotted as mean ± SEM from four biological replicates and statistics were calculated by t tests (****p < 0.0001). **c** Western blots showing the HDGF protein expression level with or without quisinostat treatment. Cells were treated by quisisnostat for 2 days and then harvested for western blotting. β-actin (ACTB) was used as standard loading control. **d** Quantification of (**c**) by ImageJ. Results were normalized to DMSO control for each cell line. Data were plotted as mean ± standard deviation from three biological replicates and statistics were calculated by t tests (****p < 0.0001). **e** TCGA analysis of the bladder cancer cohort showing that HDGF expression is higher in bladder cancer patients than in healthy tissue. Statistics were calculated by one-way ANOVA (*p < 0.05). Center lines represent the median value, upper and lower bounds of the boxes denote the upper and lower quartiles, upper and lower whiskers represent the 1.5x interquartile range, and data points outside the upper and lower whiskers are considered outliers. **f** Western blots showing that both siRNAs of HDGF (siHDGFs) can successfully knock down HDGF without affecting the expression of FGFR3 fusions in SW780 and RT112 cells. β-actin (ACTB) was used as standard loading control. C, siRNA of non-targeting control. **g**, **h** Cell viabilities of SW780 cells (**g**) and RT112 cells (**h**) treated by erdafitinib with or without HDGF knockdown. Cells were treated by erdafitinib for 3 days. And cell viabilities were determined by WST-1 assay and normalized to DMSO control for each cell line. Data were plotted as mean ± standard deviation from three biological replicates and statistics were calculated by one-way ANOVA (**p < 0.01; ****p < 0.0001). siControl, siRNA of non-targeting control. 10/25/50E, 10/25/50 nM erdafitinib; 50Q, 50 nM quisinostat.

HDACs are a series of epigenetic regulators responsible for the regulation of chromatin structure and gene transcription^27^, suggesting that FGFR3 expression level may not be the only effector that is regulated by quisinostat and mediates the synergy between erdafitinib and quisinostat. Therefore, we re-analyzed the RNA-seq data with or without quisinostat treatment. Out of the genes that were downregulated by quisinostat in all three cells (Fig. 6a), we validated that *hepatoma-derived growth factor* (*HDGF*) can also be downregulated by quisinostat at both mRNA level (Fig. 6b) and protein level (Fig. 6c, d). HDGF is an acidic heparin-binding growth factor that can function as a mitogen to promote cell proliferation and angiogenesis when secreted in the culture media^37^. It can also be localized into the nucleus in cancer cells and function as a transcription regulator^38^. Based on the analysis of a bladder cancer cohort in The Cancer Genome Atlas (TCGA) program, HDGF expression is significantly higher in bladder cancer patients compared to healthy tissue (Fig. 6e). In addition, HDGF is also overexpressed in our bladder cancer cells, compared to the healthy TRT-HU-1 cells (Supplementary Fig. 9). Noticeably, HDGF expression level is only downregulated by quisinostat, and erdafitinib does not alter HDGF expression (Supplementary Fig. 10), which indicates that HDGF may mediate the synergy independent of FGFR signaling.

In order to determine whether HDGF downregulation by quisinostat can mediate the synergy of the combination, we knocked down HDGF by siRNAs, and both siRNAs for HDGF can successfully knock down HDGF in SW780 and RT112 cells without affecting FGFR3 expression (Fig. 6f). In accordance with previous studies^38^, HDGF knockdown can suppress the proliferation of SW780 and RT112 cells (Supplementary Fig. 11A). We hypothesized that if HDGF downregulation can mediate the synergy of the combination, knocking down HDGF by siRNA should also sensitize the cells to erdafitinib. As shown in Fig. 6g, h, compared to siRNA control, erdafitinib can achieve stronger inhibitory effects after HDGF knockdown in SW780 and RT112 cells, suggesting that HDGF downregulation can sensitize BC cells to erdafitinib. In addition, HDGF knockdown does not affect Akt and Erk signaling (Supplementary Fig. 11b), which further corroborates that HDGF downregulation may mediate the synergy independent of FGFR signaling. Therefore, HDGF downregulation by quisinostat might be another mechanism behind the synergy of erdafitinib and quisinostat.

### Erdafitinib and quisinostat are synergistic in BC cells with FGFR3 activating mutations

**Fig. 7:**
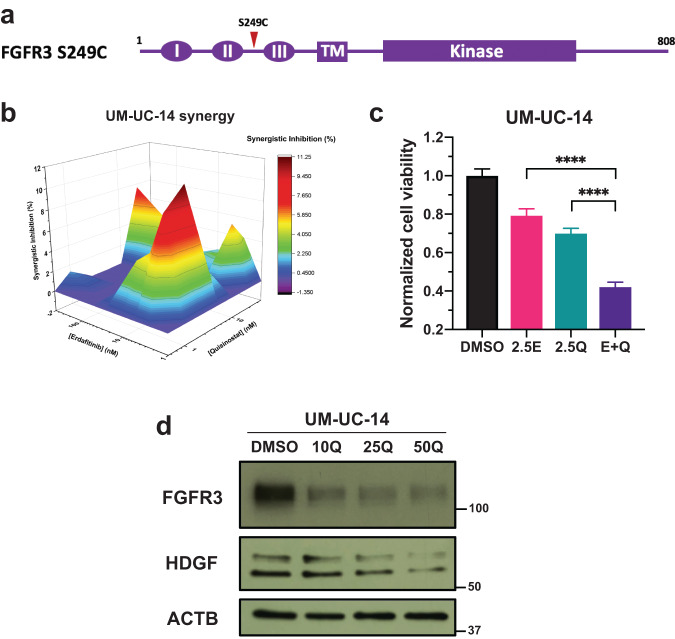
Erdafitinib and quisinostat are synergistic in BC cells with FGFR3 activating mutations. **a** Schematic diagram of FGFR3 S249 mutation. Red arrowhead represents the position of S249C in FGFR3. **b** MacSynergy II calculation (95% confidence interval) of the synergy between erdafitinib and quisinostat in UM-UC-14 cells, which is a BC cell line with FGFR3 S249C mutation. Cells were treated by different concentrations of erdafitinib and/or quisinostat for 3 days. And cell viabilities were determined by WST-1 assay and normalized to DMSO control. Synergistic inhibition was calculated by MacSynergy II. Synergistic inhibition above 0 means synergy, equal to 0 indicates additivity, and below 0 suggests antagonism. **c** Cell viability of UM-UC-14 cells by the treatment of erdafitinib and/or quisinostat. Cells were treated by erdafitinib and/or quisinostat for 3 days. And cell viabilities were determined by WST-1 assay and normalized to DMSO control. Data were plotted as mean ± standard deviation from three biological replicates and statistics were calculated by one-way ANOVA (****p < 0.0001). 2.5E, 2.5 nM erdafitinib; 2.5Q, 2.5 nM quisisnostat. **d** Western blots showing the FGFR3 and HDGF expression level with or without quisinostat treatment in UM-UC-14 cells. Cells were treated by quisisnostat for 2 days and then harvested for western blotting. β-actin (ACTB) was used as standard loading control. 10/25/50Q, 10/25/50 nM quisisnostat.

FGFR3 activating mutations are also particularly common in BC patients, and one of the most prevalent FGFR3 point mutations is FGFR3 S249C (Fig. 7a)^14^. FGFR3 S249C can self-dimerize through the formation of disulfide bond, which can lead to the constitutive activation of FGFR signaling and therefore, is regarded as an onco-driving mutation^39^. In order to extend the application of our combination of erdafitinib and quisinostat, we further validated the synergy of this combination in UM-UC-14 cells, which is a BC cell line with FGFR3 S249C mutation. Similar to FGFR3 fusion-positive BC, erdafitinib and quisinostat are also synergistic in UM-UC-14 cells (Fig. 7b), and the combination can also achieve stronger inhibition in cell viability, compared to each individual drug treatment (Fig. 7c). Furthermore, combining erdafitinib with quisinostat can also reduce the IC_50_ of quisinostat by ~60% (Table 1). In addition, quisinostat can also downregulate the expression level of FGFR3 and HDGF in UM-UC-14 cells (Fig. 7d), which further corroborates with our aforementioned results that quisinostat may achieve its synergy with erdafitinib by downregulating FGFR3 and HDGF.

## Discussion

In this study, we first confirmed the synergistic combination of erdafitinib and quisinostat in BC with FGFR3 fusions both in vitro and in vivo. Then we further investigated the molecular mechanisms behind the synergy and also validated the synergy of this combination in BC cells with FGFR3 activating mutations.

HDAC overexpression has been detected in a broad range of cancers and is often associated with poor outcomes and shorter survival, including gastric cancer, ovarian cancer, prostate cancer, and multiple myeloma, etc.^19–21,40^. In fact, vorinostat was the first HDACi approved by FDA in 2006 for cutaneous T-cell lymphoma^27^. More recently, FDA approved belinostat in 2014 for peripheral T-cell lymphoma and panobinostat in 2015 for multiple myeloma^27^. HDAC overexpression has also been reported in bladder cancer^22–24^. However, HDACi as monotherapy seems to only have limited effects for BC patients^27^. Thus, the merits of the synergy between erdafitinib and quisinostat demonstrated in this study can be of great value as a potential treatment option for BC patients with FGFR aberrations.

Takamura and colleagues explored the synergy between BGJ398, another FGFRi, and OBP-801, another HDACi^41^. However, they only studied the combination in BC cell lines without in vivo studies, and the cell lines they used lack any FGFR3 genomic alterations or overexpression. Furthermore, they used very high concentrations of BGJ398 in their studies^42^. These caveats make the practical application of the combination of FGFRi and HDACi difficult.

In our study, we validated the synergy between erdafitinib and quisinostat at low nanomolar concentrations in vitro and with dosages much lower than in previous reports in vivo, using BC cells and xenografts with FGFR3 fusions. It is well known that HDACi have relatively narrow therapeutic windows and therefore, their usage is often limited by their toxicities^27,33,34^. We also noticed possible toxicities at higher dosages of erdafitinib and quisinotat in vivo. However, when both drugs were administrated at lower dosages, we observed no apparent toxicity and the anti-tumor effects were significantly enhanced by the combinational treatment, compared to each individual drug. Similar results can also be observed in the healthy TRT-HU-1 cells. Even though quisinostat can decrease cell viability at higher concentrations, erdafitinib doesn’t significantly affect cell viability and combining erdafitinib with quisinostat doesn’t potentiate the cytotoxicity of quisinostat. These data further emphasize the value of our combinational treatment. With the combination of erdafitinib and quisinostat as a therapeutic modality, the toxicity from quisinostat can be reduced while retaining the more potent anti-tumor effects.

We next sought to understand the underlying mechanisms behind the synergy. We first revealed that quisinostat can downregulate FGFR3 expression. Interestingly, this is somewhat in contrast to our previous findings in cholangiocarcinoma with FGFR2 fusions, in which we found that quisinostat can upregulate the activation of FGFR and Erk signaling^28^. The exact mechanisms underlying these differences are currently under investigation. It is plausible that the downregulation of FGFR3 by quisinostat we observed in this study may be either an FGFR isoform specific (FGFR3 vs. FGFR2) or a cancer type specific (bladder cancer vs. cholangiocarcinoma) effect.

We also demonstrated that quisinostat can downregulate FGFR3 expression by inhibiting FGFR3 translation. It has been reported that HDACi can suppress protein translation through multiple mechanisms. Kawamata and colleagues showed that vorinostat can inhibit cyclin D1 translation by inhibiting PI3K pathway^43^. Emmrich et al. also demonstrated that vorinostat can increase the level of microRNA-139–5p, which can downregulate translation initiation factor eIF4G2 and therefore, lead to the inhibition of protein translation^44^. Our RNA-seq results also revealed the downregulation of several translation initiation factors and regulators by quisinostat (Supplementary Fig. 12). While the exact mechanism of how quisinostat inhibits FGFR3 translation is still currently under investigation, nevertheless, our data indicates a new promising strategy of combining FGFR inhibitors with drugs that can inhibit FGFR expression or facilitate FGFR degradation to enhance the efficiency and prevent/overcome drug resistance for BC treatment.

We also revealed a second mechanism behind the synergy of erdafitinib and quisinostat: quisinostat can sensitize BC cells to erdafitinib by downregulating HDGF. HDGF is an acidic heparin-binding growth factor that was first discovered in the conditioned media of a human hepatoma cell line, Huh-7^45^. It was initially regarded as a mitogen that can promote cell proliferation, migration, and angiogenesis, etc.^45^. It was later discovered to also localize in nucleus in cancer cells to function as a transcription regulator by binding to DNA through its PXXP domain^38^. HDGF overexpression and its prognostic value has been reported in a number of cancers^46–49^. Based on the analysis of the bladder cancer cohort from TCGA and some previous studies^38^, HDGF is also overexpressed in bladder cancer. Overexpression of HDGF has been reported to promote cell proliferation, migration, and invasion in bladder cancer^38^.

In our study, we demonstrated that HDGF knockdown can sensitize SW780 and RT112 cells to erdafitinib, suggesting that quisinostat-mediated HDGF downregulation might be another mechanism behind the synergy. In addition, HDGF knockdown doesn’t affect FGFR3 expression and Erk and Akt signaling activation, indicating that HDGF downregulation by quisinostat might be a mechanism independent of FGFR3 signaling. HDGF has been reported to be related to multiple signaling pathways and biological processes, including PKC, NF-kB, STAT3, p38, and glycolysis^50–53^. Therefore, dissecting the molecular relationship between HDGF and erdafitinib sensitivity is still an ongoing work-in-progress. These results suggest the possibility of developing HDGF inhibitors for BC treatment or using HDGF as a biomarker and open up new opportunities for combinational targeted therapy of FGFR and HDGF inhibitors.

Other than FGFR3 fusion-positive BC, we also verified that erdafitinib and quisinostat are synergistic in UM-UC-14 bladder cancer cells with FGFR3 S249C activating mutation, which is one of the most prevalent FGFR3 point mutations in BC. And we also demonstrated that FGFR3 and HDGF can be downregulated by quisinostat treatment in UM-UC-14 cells. These data suggest that the synergy between erdafitinib and quisinostat has a broader application in BC patients with FGFR3 genomic alterations, including FGFR3 fusions and activating mutations.

In conclusion, in this study, we first confirmed the synergy between erdafitinib and quisinostat in BC cells with FGFR3 fusions in vitro. Then we further demonstrated their combinational benefits in BC xenografts with FGFR3 fusions in vivo. To understand the molecular mechanisms behind the synergy, we revealed that quisinostat can downregulate FGFR3 expression by suppressing FGFR3 translation. In addition, quisinostat can also downregulate HDGF, which can sensitize BC cells to erdafitinib. These innovative mechanistic insights investigated in this study can contribute to the future development of new therapies and facilitate the revelation of new prognosis biomarkers for BC patients with FGFR3 aberrations. To demonstrate the generality of this combination, we also verified the synergy between erdafitinib and quisinostat in BC cells with FGFR3 activating mutations. Our results provide the preclinical proof of concept for the translation of the combination of erdafitinib and quisinostat from preclinical studies into clinical trials for the treatment of BC patients with FGFR aberrations.

## Methods

### Cell lines and culture conditions

SW780 cells were purchased from ATCC (#CRL-2169) and RT112 cells were purchased from ECACC (#85061106). RT4 cells were kindly provided by Dr. Darryl Martin’s lab (Yale University, New Haven, CT, USA). UM-UC-14 cells were a generous gift from Dr. Sharada Mokkapati’s lab (MD Anderson Cancer Center, Huston, TX, USA). TRT-HU-1 cells were kindly provided by Dr. Rosalyn M. Adam (Harvard Medical School, Boston, MA, USA). SW780 cells were cultured in DMEM media (Gibco) supplied with 10% heat-inactivated FBS (HIFBS, Gibco) and 1% antibiotic-antimycotic (A/A, Gibco). RT112 cells were maintained in DMEM media supplied with 10% HIFBS, 1% A/A, 1% non-essential amino acid (NEAA, Gibco), and 2 mM L-glutamine (Gibco). RT4 cells were cultured in McCoy’s 5A (ATCC) media supplied with 10% heat-inactivated FBS (HIFBS) and 1% antibiotic-antimycotic (A/A). UM-UC-14 cells were maintained in DMEM media supplied with 10% HIFBS, 1% A/A, 1% NEAA, 2 mM L-glutamine, and 1 mM sodium pyruvate (Gibco). TRT-HU-1 cells were cultured in DMEM media supplied with 15% non-heat inactivated FBS (Gibco), 1% A/A, 1% NEAA, 110 mg/L sodium pyruvate (Gibco), 1.15 mM 1-thioglycerol (Sigma), and 2 mM L-glutamine (Gibco). All cells were cultured at 37 °C in a humidified atmosphere with 5% CO_2_ and tested to be mycoplasma negative.

### Chemicals and antibodies

Erdafitinib (#HY-18708) and quisinostat (#HY-15433) were purchased from MedChemExpress. L-methionine was purchased from Sigma-Aldrich (#M5308).

The following primary antibodies were purchased from Cell Signaling Technology (CST): phospho-FGFR (#3476), phospho-FRS2α Y196 (#3864), Akt (#4691), phospho-Akt S473 (#4060), Erk1/2 (#4695), phospho-Erk1/2 T202/Y204 (#4370), HDGF (#42105), β-actin (#4967), EGFR (#4267), HDAC1 (#34589), HDAC2 (#57156), HDAC3 (#85057), ErbB2 (#4290), ErbB3 (12708), ErbB4 (#4795), and GAPDH (#3683). FRS2α primary antibody (#MAB4069) is purchased from R&D Systems. FGFR3 primary antibody (#sc-13121) is purchased from Santa Cruz Biotechnology. HRP-linked rabbit IgG secondary antibody (#7074) and HRP-linked mouse IgG secondary antibody (#7076) are purchased from CST.

### Cloning and cell line engineering

FGFR3-TACC3 cDNA was extracted from RT112 cells using the following forward primer with a Xba I site and reverse primer with a Not I site:

FGFR3 For: 5′-AAGTAAtctagaGCCACCATGGGCGCCCCTGCCTGCGCC-3′

TACC3 Rev: 5′-GGACGTgcggccgcTCAGATCTTCTCCATCTTGGAGATG-3′

Briefly, RNA was first extracted from RT112 cells by RNeasy Mini Kit (QIAGEN, #74104), and then cDNA was synthesized using SuperScript IV First-Strand Synthesis System (Invitrogen, #18091050). FGFR3-TACC3 cDNA was then amplified by PCR using Platinum SuperFi II DNA polymerase (Invitrogen, #12361010).

The PCR products were then cloned into pCDH-CMV-MCS-EF1-puro vectors (SBI, #CD510B-1) using Xba I (NEB, #R0145) and Not I (NEB, #0189) restriction enzymes.

For the lentivirus packaging, dR8.91 and VsV-G plasmids are generously gifted by Dr. David Calderwood’s lab (Yale University, New Haven, CT, USA). HEK293T cells were first cultured to ~70–80% confluency in 10-cm dishes in DMEM media with 10% HIFBS. Then a mixture of 4.44 μg dR8.91, 2.22 μg VsV-G, and 4.44 μg pCDH plasmids were combined with 13.2 μg PEI in 1 ml Opti-MEM. The mixture was incubated at room temperature for 15 min before added into the HEK293T cells for transfection. The media was replaced by fresh DMEM media with 10% HIFBS 24 h after transfection. And after another 24 h, the media containing lentivirus was collected, aliquoted, flash frozen, and stored at −80 °C until use.

To establish the stable RT112 cells overexpressing FGFR3-TACC3, 1 × 10^5^ cells/well of RT112 cells were seeded in 6-well plates. The next day, the media was replaced by 2 ml/well lentivirus media with 1:1 dilution in complete culture media for virus infection. 24 h after virus infection, the media was replaced by complete culture media with 2 μg/ml puromycin for selection. After selection, cells were cultured and maintained in complete culture media with 2 μg/ml puromycin.

### Cell viability assay and synergy calculation

3 × 10^3^ cells/well of SW780 cells, 1 × 10^4^ cells/well of RT112 cells, 1 × 10^4^ cells/well of RT4 cells, 3 × 10^3^ cells/well of UM-UC-14 cells, and 5 × 10^3^ cells/well of TRT-HU-1 cells were seeded in 96-well plates (Greiner Bio-one) with a serial of concentrations of erdafitinib and quisinostat. To investigate the effects of FGF1 on the combinational treatment, 50 ng/ml of FGF1 (Gibco, #PHG0014) and 10 μg/ml of heparin (STEMCELL Technologies, #07980) were added into the cell culture media. Cells were treated for 3 days and cell viability was determined by WST-1 cell proliferation assay kit (Takara Bio, #MK400) following manufacturer’s protocol. Cell viability was normalized to 0 nM erdafitinib under different concentrations of quisinostat to calculate the effects of quisinostat on erdafitinib sensitivity. Synergy was calculated by MacSynergy II with 95% confidence interval^32^. Synergistic inhibition that is above 0 is considered as synergy, equal to 0 as additivity, and below 0 as antagonism. Data was plotted by Origin.

### Clonogenic assay

Clonogenic assay was performed as described previously with minor modifications^54^. Briefly, 1 × 10^4^ cells/well of SW780 cells, 1 × 10^4^ cells/well of RT112 cells, and 4 × 10^4^ cells/well of RT4 cells were seeded in 6-well plates (Corning) with indicated concentrations of erdafitinib and quisinostat. Cells were treated for 9 days. After treatment, cells were first fixed by 4% formaldehyde (Thermo Scientific, #28908) for 15 min and then stained by 0.5% crystal violet (Sigma-Aldrich, #192-12) for 45 min. Cells were then washed by deionized water, air-dried, and imaged. Colony area was quantified by ImageJ and plotted by GraphPad Prism (version 8.1.0).

### Animal studies

All animal studies were performed in accordance with Yale institutional animal care and use committee (IACUC) approved protocol - CPCM - 2021-20218.

1 × 10^7^ cells of SW180 or RT112 cells were implanted subcutaneously into the right flank of immune deficient Rag2/IL2RG double knockout mice (Envigo) in the presence of 50% Matrigel (Corning). For combinational treatment studies, mice with palpable and similar sized tumors between 3–7 days post-implantation were randomized to four experimental groups: vehicle, erdafitinib, quisinostat, and combinational treatment. The dosage of 10 mg/kg of erdafitinib was selected for both xenografts and quisinostat was dosed at 10 mg/kg for SW780 tumors and 5 mg/kg for RT112 tumors. The dose administration was tapered down to once every 3 days dosing after about 2 weeks of everyday administration upon observation of possible toxicity indicated by reduced activity of some animals. Both drugs were formulated in 20% 2-hydroxy propyl ß-cyclodextrin and administered in 100 μl volumes via the oral route by gavage for erdafitinib and intraperitoneal injection for quisinostat. Tumor volumes were recorded by caliper measurements at 3-day intervals and tumor volumes were calculated using the formula—½ × length × width^2^. The mice were removed from the study and euthanized upon reaching an endpoint volume of 1000 mm^3^ in accordance with the IACUC protocol. Briefly, the mice were placed in an anesthesia chamber of RAS4 rodent anesthesia system (Kent Scientific, CT), administered an overdose of isoflurane and death confirmed by cessation of respiratory movements and heartbeat for at least 30 s. Log-Rank (Mantel-Cox) test was used to test for significance in survival rates of the treatment groups.

### Western blotting

The effects of erdafitinib and/or quisinostat on cell signaling and protein expression were examined by western blot as described previously with minor modifications^28^. Briefly, to determine the effects of erdafitinib and/or quisinostat on FGFR signaling, cells were first treated by quisinostat for 2 days, followed by the erdafitinib treatment for 3 h or 1 day and no serum starvation for 3 h. DMSO was used as treatment control. 50 ng/ml of FGF1 and 10 μg/ml of heparin were then added into the cells and incubated for 15 min in cell incubator to stimulate FGFR signaling. Then cells were lysed by RIPA lysis buffer (Millipore), supplied with 0.1% SDS, protease inhibitor cocktail (Roche), 1 mM sodium orthovanadate, 2 mM beta-glycerophosphate, 25 mM sodium fluoride, 1 mM sodium pyrophosphate. Cell lysates were then cleared by centrifuging at 15,000 rpm, 4 °C for 10 min.

To the investigate the effects of erdafitinib and/or quisinostat on protein expression, cells were first treated by indicated concentrations of erdafitinib and/or quisinostat for 2 days. Then cells were lysed and cell lysates were prepared as described above.

Same amount of protein lysate (5–20 μg) was separated by SDS-PAGE and transferred onto nitrocellulose membrane. The membrane was blocked by 5% non-fat milk and then incubated with primary antibodies at 4 °C overnight. Membrane was then washed by 1x TBST and incubated with HRP-conjugated anti-mouse or anti-rabbit secondary antibodies, depending on the primary antibodies. All primary and secondary antibodies were used at 1:1000 dilution. The blots were then developed by SuperSignal™ West Femto Maximum Sensitivity Substrate (Thermo Fisher Scientific, #34095) and exposed to X-ray film. GAPDH or β-actin (ACTB) were used as standard loading control. Unprocessed blots are provided in Supplementary Fig. 13.

### RT-qPCR

Cells were first treated by quisinostat or DMSO control for 2 days in 6-well plates. Total RNA was then extracted by RNeasy Mini Kit (QIAGEN, #74104) and cDNA was synthesized by iScript cDNA Synthesis kit (Bio-Rad, #170-8891), according to manufacturers’ protocols. qPCR was then performed with iTaq Universal SYBR Green Supermix (Bio-Rad, #1725121) following manufacturer’s protocol, using the primers below:

FGFR3 Primer 1: 5′-GTACTGTGCCACTTCAGTGT-3′

FGFR3 Primer 2: 5′-CCAGCAGCTTCTTGTCCATC-3′

HDGF Primer 1: 5′-ACAACCCTACTGTCAAGGC-3′

HDGF Primer 2: 5’-TCTTATCACCGTCACCCTCTG-3’

ACTB Primer 1: 5′-ACAGAGCCTCGCCTTTG-3′

ACTB Primer 2: 5′-CCTTGCACATGCCGGAG-3′

ACTB was used as standard loading control. All primers were purchased from Integrated DNA Technologies (IDT).

### RNA-seq analysis

Cells were first treated by 10–50 nM quisinostat or DMSO control for 2 days in 6-well plates. Total RNA was then extracted by TRIZol Plus RNA Purification kit (Invitrogen, #12183555) following manufacturer’s protocol. RNA-seq was then performed by NovaSeq 6000 (Illumina), and the results were analyzed by the HISAT2 – StringTie – Ballgown pipeline as described previously^55^. Gene ontology enrichment analysis was performed through Gene Ontology Resource (http://geneontology.org/). An unbiased presentation of the RNA-seq results is provided in Supplementary Dataset 1.

### Protein translational activities

Cells were first treated by quisinostat or DMSO control for 2 days in 6-well plates, followed by the starvation of no L-methionine DMEM media (Gibco, #21013024) overnight.

Total protein translational activities were determined by Click-iT™ HPG Alexa Fluor™ 594 Protein Synthesis Assay Kit (Invitrogen, #C10429) according to manufacturer’s protocol. AF594 signal was detected by Zeiss LSM 880 Airyscan microscope or BD LSRII flow cytometer system. Microscopic data was processed by ImageJ Fiji. Flow cytometry data was analyzed by FlowJo and the gating strategy is provided in Supplementary Fig. 14. The geometric mean of each sample is normalized to DMSO control for each cell line and plotted by GraphPad Prism (version 8.1.0).

For FGFR3 translation, a final concentration of 0.2 mM L-methionine was added into the culture media after starvation and cells were incubated for another 8 h in cell incubator. Then cell lysates were prepared and subjected to western blot as described above.

### TCGA analysis

Expression data of HDGF in a bladder cancer cohort from TCGA database was accessed and analyzed by Gene Expression Profiling Interactive Analysis (GEPIA, http://gepia.cancer-pku.cn/).

### siRNA knockdown

siRNAs for FGFR3 (siFGFR3), HDGF (siHDGF), and non-targeting control (siControl) was purchased from IDT. The sequences of all siRNAs are described below. Cells were first seeded in 6-well plates overnight. The next day, siRNAs were transfected using Lipofectamine™ RNAiMAX Transfection Reagent (Invitrogen, #13778075) according to manufacturer’s protocol. siRNAs were used at following concentrations: 0.5 nM siFGFR3-1, 2.5 nM siFGFR3-2, 10 nM HDGF-1, and 10 nM HDGF-2. Cells were transfected for 2 days before subjected to western blot or cell viability assays as described above.

siControl: 5′-CGUUAAUCGCGUAUAAUACGCGUAT-3′

5′-AUACGCGUAUUAUACGCGAUUAACGAC-3′

siFGFR3-1: 5′-GUGGAGCCUGGUCAUGGAAAGCGTG-3′

5′-CACGCUUUCCAUGACCAGGCUCCACUG-3′

siFGFR3-2: 5′-GACCGAGGACAACGUGAUGAAGATC-3′

5′-GAUCUUCAUCACGUUGUCCUCGGUCAC-3′

siHDGF-1: 5′-UCCCUUACGAGGAAUCCAAGGAGAA-3′

5′-UUCUCCUUGGAUUCCUCGUAAGGGAAG-3′

siHDGF-2: 5′-ACUGUCAAGGCUUCCGGCUAUCAGT-3′

5′-ACUGAUAGCCGGAAGCCUUGACAGUAG-3′

### Ethics approval

All animal studies were performed in accordance with Yale institutional animal care and use committee (IACUC) approved protocol - CPCM - 2021-20218.

### Statistical analysis

Data were plotted as means ± SD or means ± SEM from at least three biological replicated experiments, as indicated in the corresponding figure legends. Statistical analysis was calculated by t tests or one-way ANOVA as indicated in the corresponding figure legends, using GraphPad Prism (version 8.1.0). The statistical significance is annotated as follows: ns, non-significant; *P ≤ 0.05; **P ≤ 0.01; ***P ≤ 0.001; ****P ≤ 0.0001. Differences with *P* ≤ 0.05 are considered statistically significant.

### Reporting summary

Further information on research design is available in the Nature Research Reporting Summary linked to this article.

## Supplementary information

Supplementary figures

Dataset 1

REPORTING SUMMARY

## Data Availability

The RNA-seq data generated in this study are publicly available in Gene Expression Omnibus (GEO) at GSE214814. The TCGA database for the bladder cancer cohort can be accessed and analyzed by Gene Expression Profiling Interactive Analysis (GEPIA, http://gepia.cancer-pku.cn/). The rest of the data generated in this study are available within the article and its supplementary data files.

## Code availability

The underlying code for RNA-seq analysis in this study is available in GitHub repository and can be accessed via this link https://github.com/ZechenWangKSAYale/Bladder-cancer-combinational-therapy/tree/bf1fb488083305f6bd780cf3a5e9514d0c6d31c3. For RNA-seq analysis, the adaptor and bad quality reads were first trimmed out by trimmomatic v0.36 using the parameter "ILLUMINACLIP:TruSeq3-PE-2.fa:2:30:10:8:TRUE LEADING:3 TRAILING:3 SLIDINGWINDOW:4:15 MINLEN:36". Then all reads were mapped and aligned to Ensembl GRCh38 using HISAT2 v2.2.1. The mapped and aligned reads were then sorted by samtools v1.15.1. Next, the transcriptome was assembled and gene expression abundance was calculated by stringtie v2.2.1. Finally, FKPM of each gene for each sample was calculated by Ballgown v2.26.0.
