# Supplementary material for: Tackling FGFR3-driven bladder cancer with a promising synergistic FGFR/HDAC targeted therapy
Source: NPJ Precis Oncol. 2023 Jul 21;7:70. doi: 10.1038/s41698-023-00417-5 (PMC10362036; doi:10.1038/s41698-023-00417-5)
Supplement: Supplementary file 1 — Supplementary figures [file 41698_2023_417_MOESM1_ESM.pdf]

# Supplementary Figures

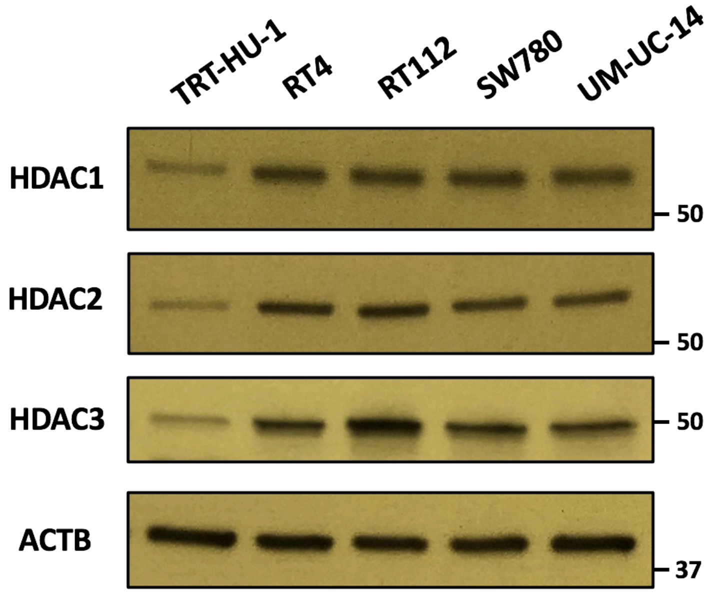


**Supplementary Figure 1. HDACs are overexpressed in bladder cancer cells.**
Western blots showing the expression level of HDAC1/2/3 in bladder cancer cells, compared to healthy bladder epithelial cells. TRT-HU-1 is the immortalized healthy human bladder epithelial cells. RT4, RT112, and SW780 are bladder cancer cells with FGFR3 fusions. UM-UC-14 is bladder cancer cells with FGFR3 S249C activating mutation. β-actin (ACTB) was used as standard loading control.

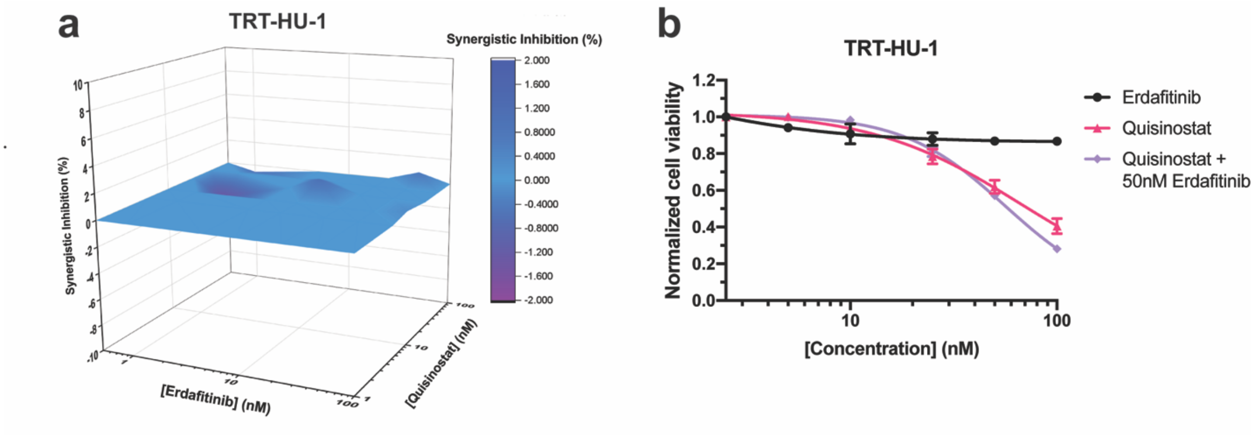


**Supplementary Figure 2. Erdafitinib and quisinostat are additive in TRT-HU-1 cells.**
**a,** MacSynergy II calculation (95% confidence interval) of the combinational effects of erdafitinib and quisinostat in TRT-HU-1 cells. Synergistic inhibition above 0 means synergy, equal to 0 indicates additivity, and below 0 suggests antagonism. **b,** Dose-response curves of erdafitinib and quisinostat in TRT-HU-1 cells. Cells were treated by erdafitinib and/or quisinostat for 3 days, and cell viabilities were determined by WST-1 assays. All data were normalized to DMSO control (for erdafitinib or quisinostat individual treatment) or no erdafitinib control (for quisinostat and erdafitinib combinational treatment). Each data point was plotted as mean ± standard deviation from three biological replicates.

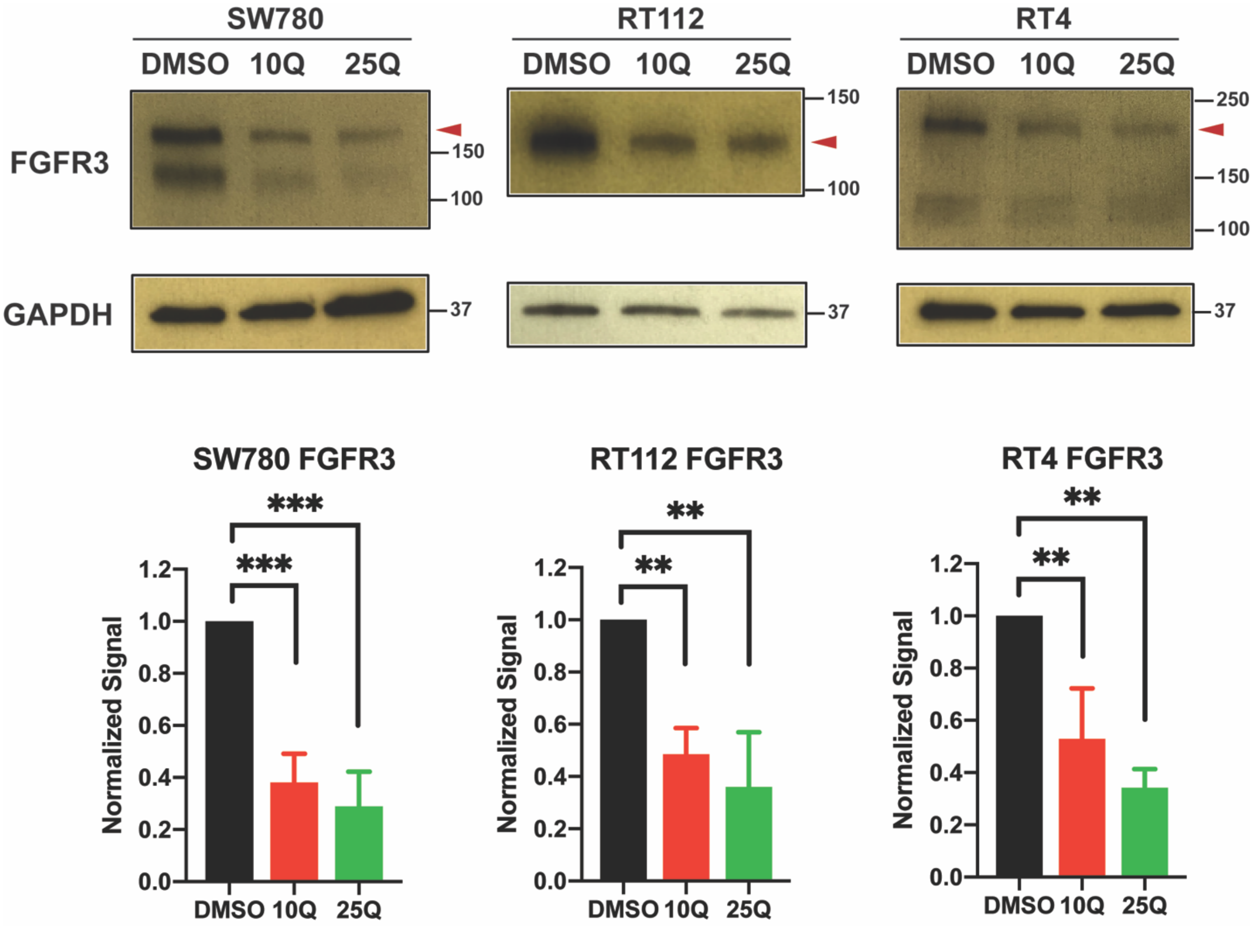


**Supplementary Figure 3. Quisinostat treatment can downregulate FGFR3 expression level.** Western blots showing that quisinostat treatment can downregulate the expression level of FGFR3 in SW780, RT112, and RT4 cells. The positions of FGFR3 fusions are indicated by the red arrowhead. GAPDH was used as standard loading control. Results were quantified by ImageJ and normalized to DMSO control. Data are plotted as mean ± standard deviation from three biological replicates and statistics are calculated by one-way ANOVA (**, $p<0.01$; ***, $p<0.001$).

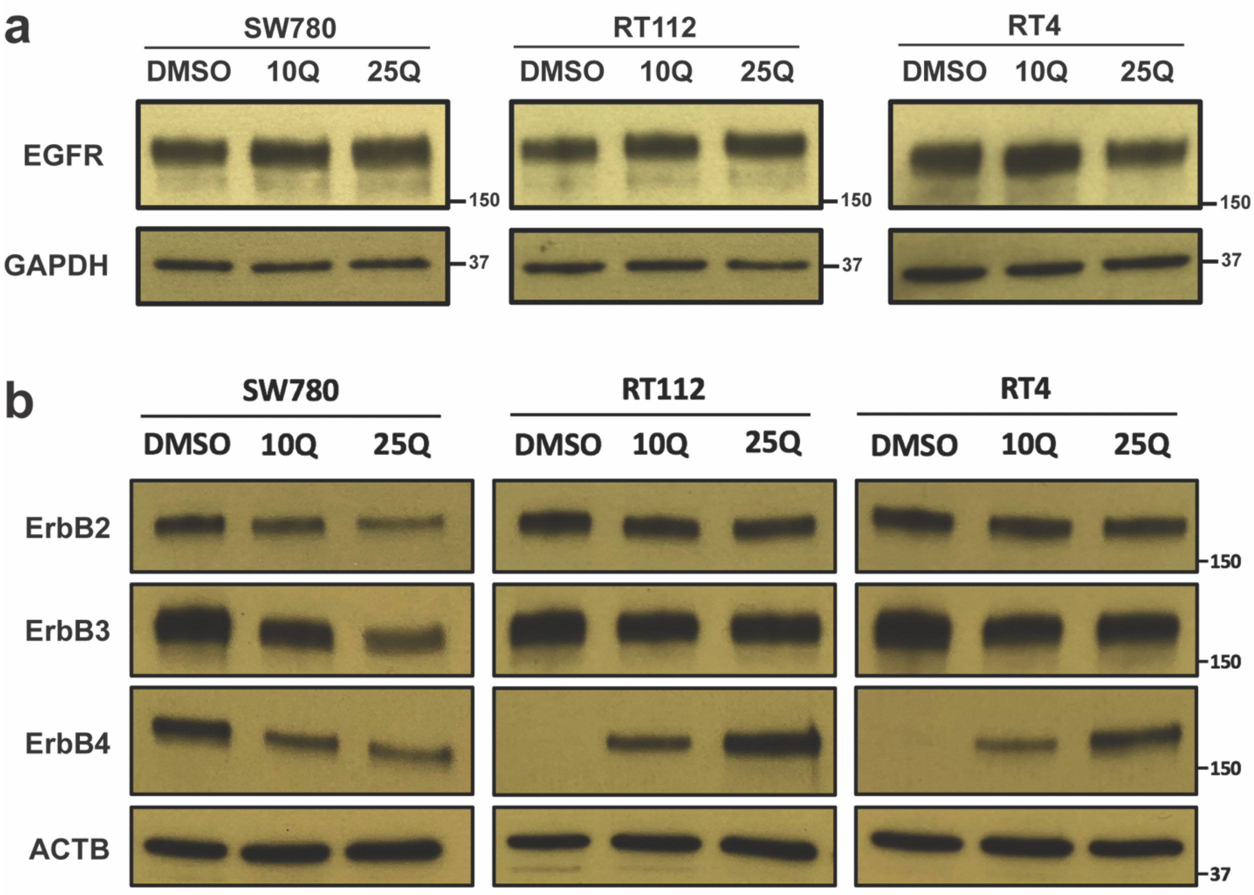


**Supplementary Figure 4. Effects of quisinostat treatment on EGFR/ErbB expression.**
**a,** Western blots showing the effects of quisinostat treatment on the expression level of EGFR in SW780, RT112, and RT4 cells. GAPDH was used as standard loading control. **b,** Western blots showing the effects of quisinostat treatment on the expression level of ErbB2/3/4 in SW780, RT112, and RT4 cells. β-actin (ACTB) was used as standard loading control. Cells were treated by quisinostat for 2 days and harvested for western blotting. 0/10/25Q, 0/10/25nM quisinostat treatment.

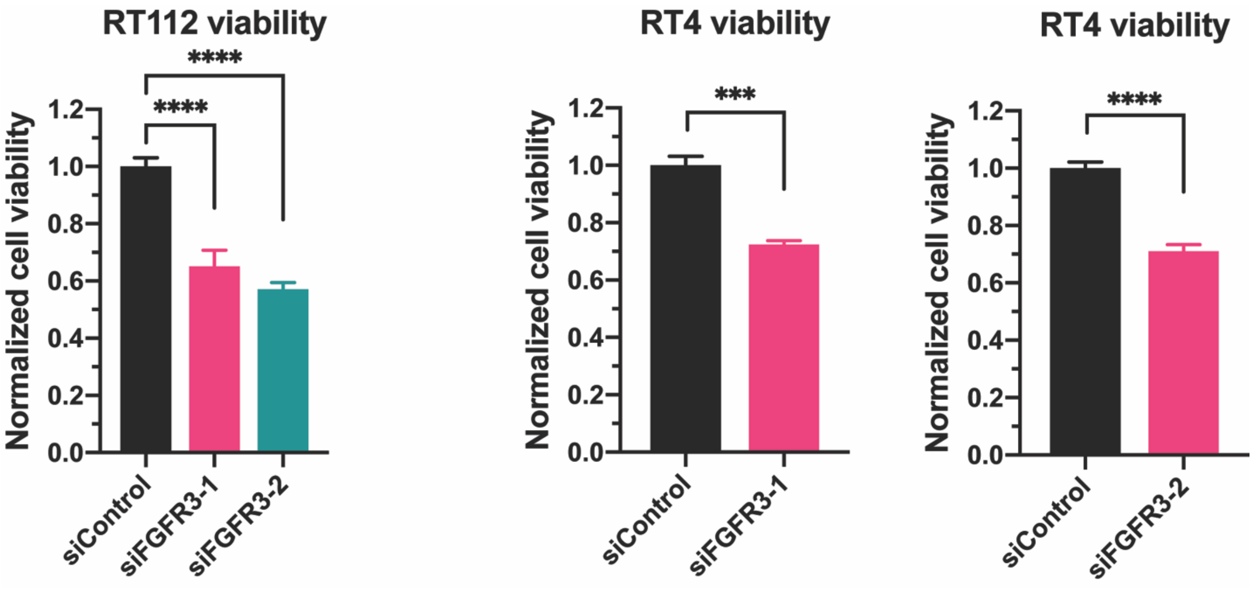


**Supplementary Figure 5. Knocking down FGFR3 decreases cell viability of RT112 and RT4 cells.**

Cells were first treated by siRNA for FGFR3 (siFGFR3) or control (siControl) for 2 days. Then cells were seeded in 96-well plates and cultured for 3 days. Cell viability was then determined by WST-1 assay. All data were normalized to siControl. Data are plotted as mean ± standard deviation from three biological replicates and statistics are calculated by one-way ANOVA (for RT112 cells) or t tests (for RT4 cells) (***, $p<0.001$; ****, $p<0.0001$).

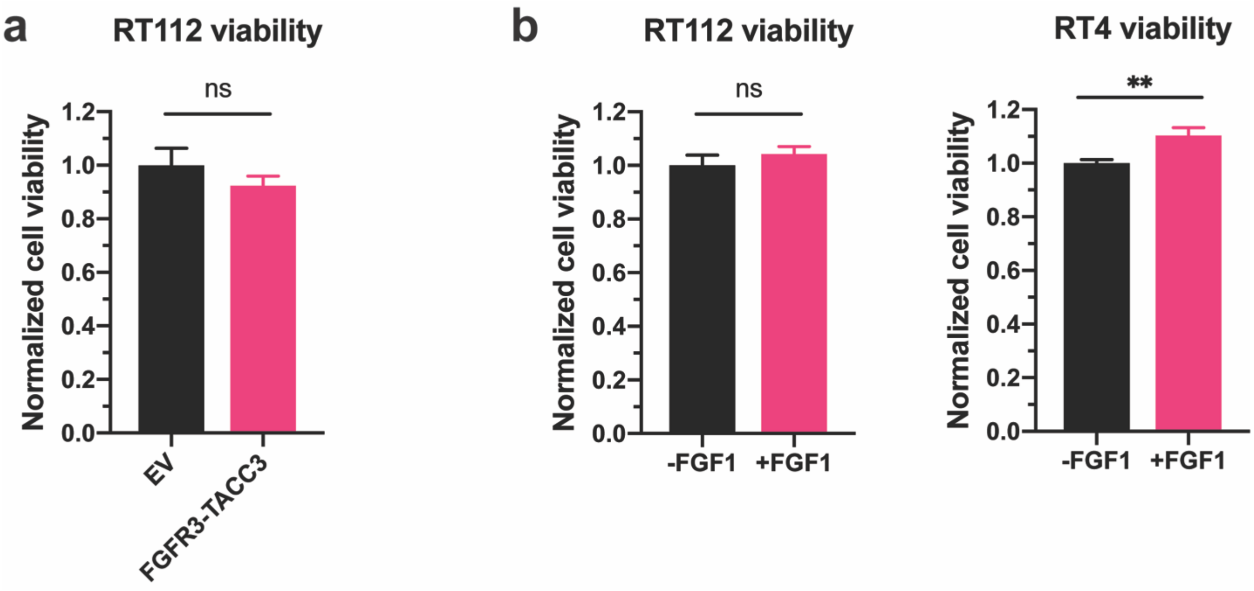


**Supplementary Figure 6. Effects of FGFR3-TACC3 overexpression and FGF1 treatment on cell viabilities.**
**a,** Cell viabilities of RT112 cells overexpressing FGFR3-TACC3 or empty vector (EV). Cells were seeded in 96-well plates and cultured for 3 days. Cell viability was then determined by WST-1 assay. All data were normalized to EV. Data are plotted as mean ± standard deviation from three biological replicates and statistics are calculated by t tests. **b,** Cell viabilities of RT112 and RT4 cells treated with or without FGF1. Cells were seeded in 96-well plates with or without 50ng/ml FGF1 + 10µg/ml heparin and cultured for 3 days. Cell viability was then determined by WST-1 assay. All data were normalized to no FGF1 control. Data are plotted as mean ± standard deviation from three biological replicates and statistics are calculated by t tests. (ns, non-significant; **, $p<0.01$).

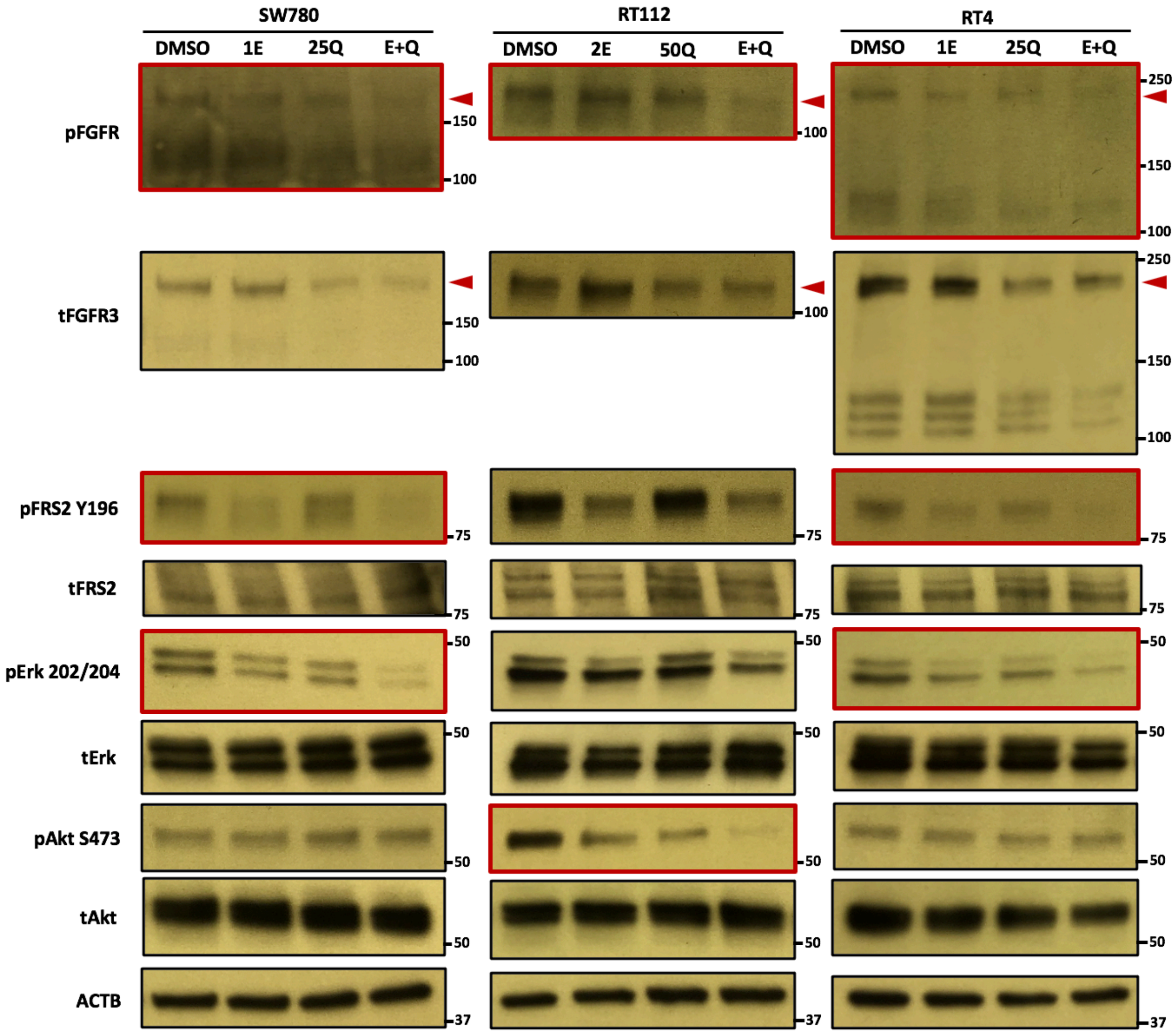


**Supplementary Figure 7. Quisinostat can also concomitantly inhibit FGFR signaling with long-term erdafitinib treatment.**

Cells were first treated by quisinostat or DMSO control for 2 days, followed by the treatment by erdafitinib or DMSO control for 1 day and then 3 h starvation with no serum media. Then FGFR signaling was stimulated by 50ng/ml FGF1 + 10μg/ml heparin for 15min and analyzed by western blotting. The positions of FGFR3 fusions are indicated by the red arrowhead. β-actin (ACTB) was used as standard loading control. 1E, 1 nM erdafitinib; 2E, 2 nM erdafitinib; 25Q, 25 nM quisinostat; 50Q, 50 nM quisinostat; E+Q, the combination of erdafitinib and quisinostat. Red boxes represent the signaling pathways that can be further inhibited by the combinational treatment.

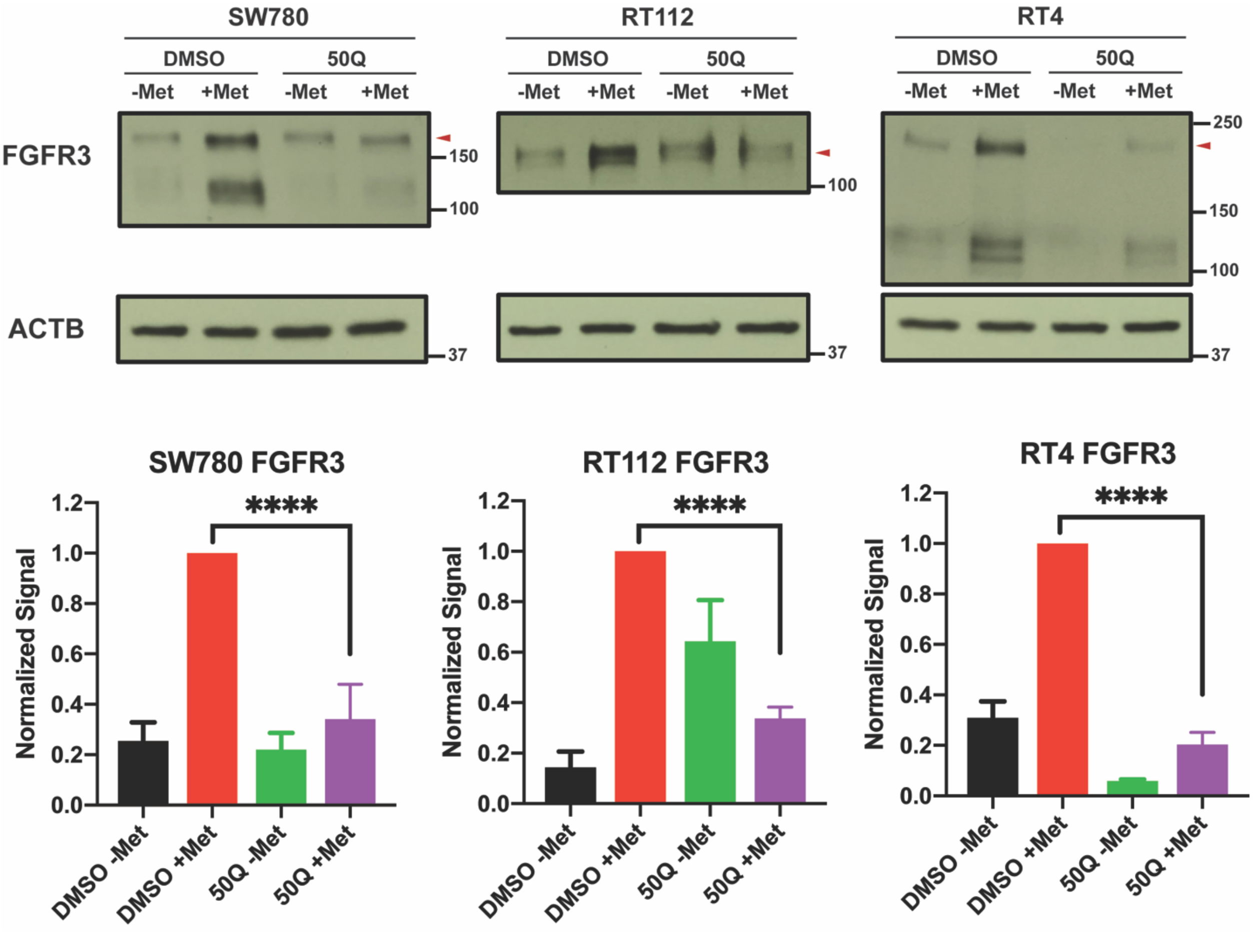


**Supplementary Figure 8. Quisinostat treatment suppresses FGFR3 protein translation.** Western blots showing the effects of quisinostat on FGFR3 protein translation. Cells were first treated by quisinostat or DMSO for 2 days and then starved by no L-methionine media overnight. L-methionine was then added into the culture media and cells were incubated for another 8 h. Then cells were lysed and analyzed by western blotting. β-actin (ACTB) was used as standard loading control. The positions of FGFR3 fusions are indicated by the red arrowhead. Results were quantified by ImageJ and normalized to DMSO +Met treatment. Data are plotted as mean ± standard deviation from three biological replicates and statistics are calculated by one-way ANOVA (****, $p<0.0001$). 50Q, 50 nM quisinostat; Met, L-methionine.

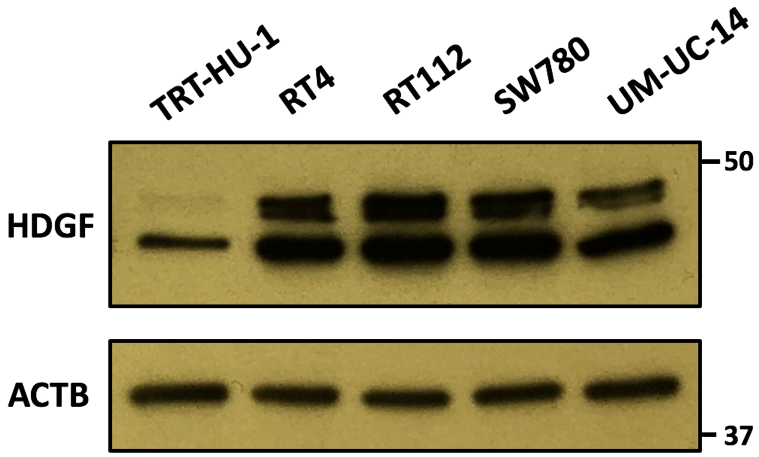


**Supplementary Figure 9. HDGF is overexpressed in bladder cancer cells.**
Western blots showing the expression level of HDGF in bladder cancer cells, compared to healthy bladder epithelial cells. TRT-HU-1 is the immortalized healthy human bladder epithelial cells. RT4, RT112, and SW780 are bladder cancer cells with FGFR3 fusions. UM-UC-14 is bladder cancer cells with FGFR3 S249C activating mutation. β-actin (ACTB) was used as standard loading control.

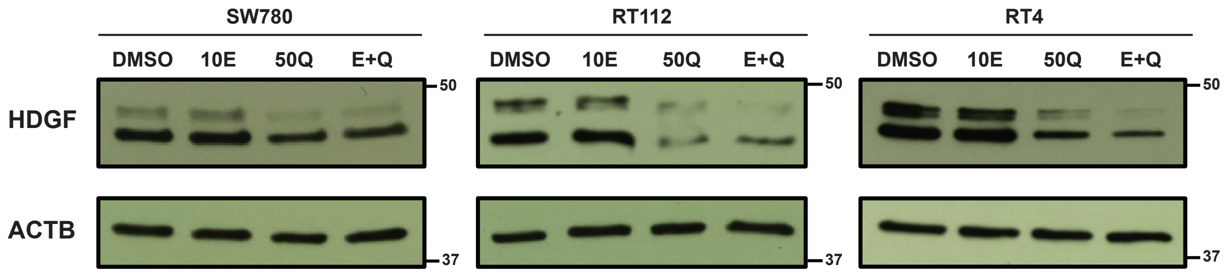


**Supplementary Figure 10. Erdafitinib treatment cannot affect HDGF expression.** Cells were treated by erdafitinib, quisinostat, or the combination for 2 days. Cells were then lysed and HDGF expression was analyzed by western blotting. β-actin (ACTB) was used as standard loading control. 10E, 10nM erdafitinib; 50Q, 50nM quisinostat; E+Q, the combination of erdafitinib and quisinostat.

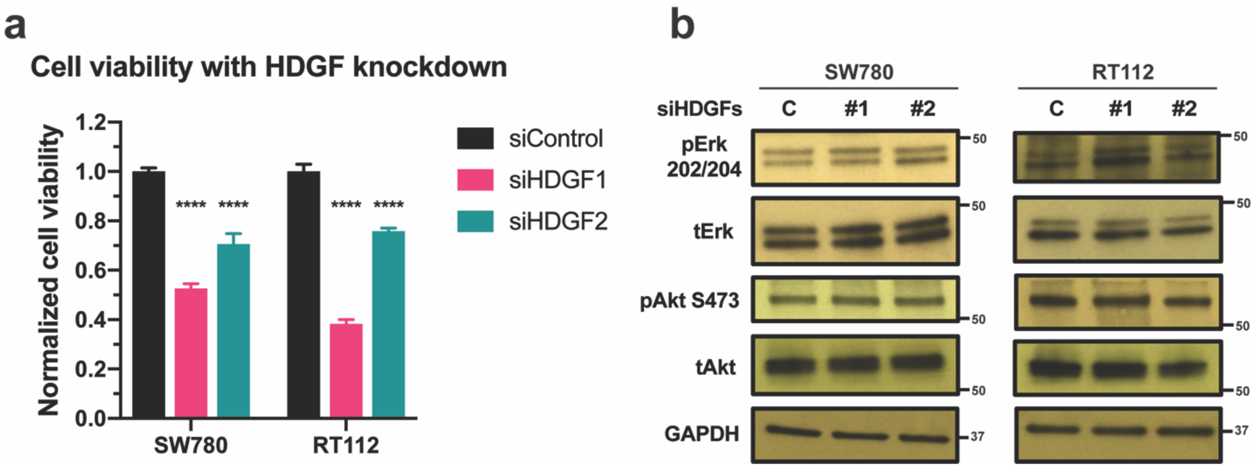


**Supplementary Figure 11. HDGF knockdown decreases cell proliferation without affecting FGFR signaling.**

**a,** Cell viabilities of SW780 and RT112 cells with HDGF knockdown. Cells were first treated by siRNA for HDGF (siHDGF) or control (siControl) for 2 days. Then cells were seeded in 96-well plates and cultured for 3 days. Cell viability was then determined by WST-1 assay. All data were normalized to siControl. Data are plotted as mean ± standard deviation from three biological replicates and statistics are calculated by one-way ANOVA (****, $p<0.0001$). **b,** Western blots showing the effects of HDGF knockdown on phospho-Erk and phospho-Akt signaling activation in SW780 and RT112 cells. Cells were first treated by siRNA for HDGF (siHDGF) or control (siControl) for 2 days. Then cells were lysed and analyzed by western blotting. GAPDH was used as standard loading control. C, siRNA of non-targeting control.

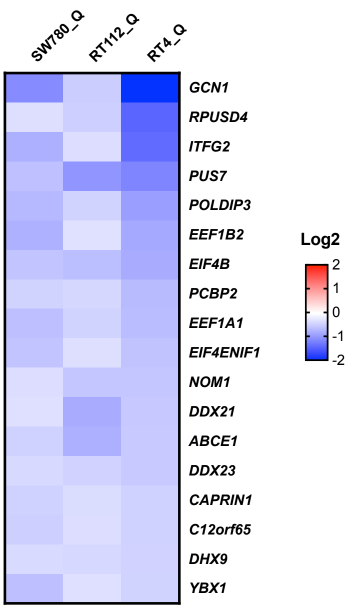


**Supplementary Figure 12. Quisinostat treatment may downregulate translation initiation factors and regulators.**
RNA-seq results showing the genes that are involved in protein translation initiation or regulation which can be downregulated by quisinostat treatment in SW780, RT112, and RT4 cells.

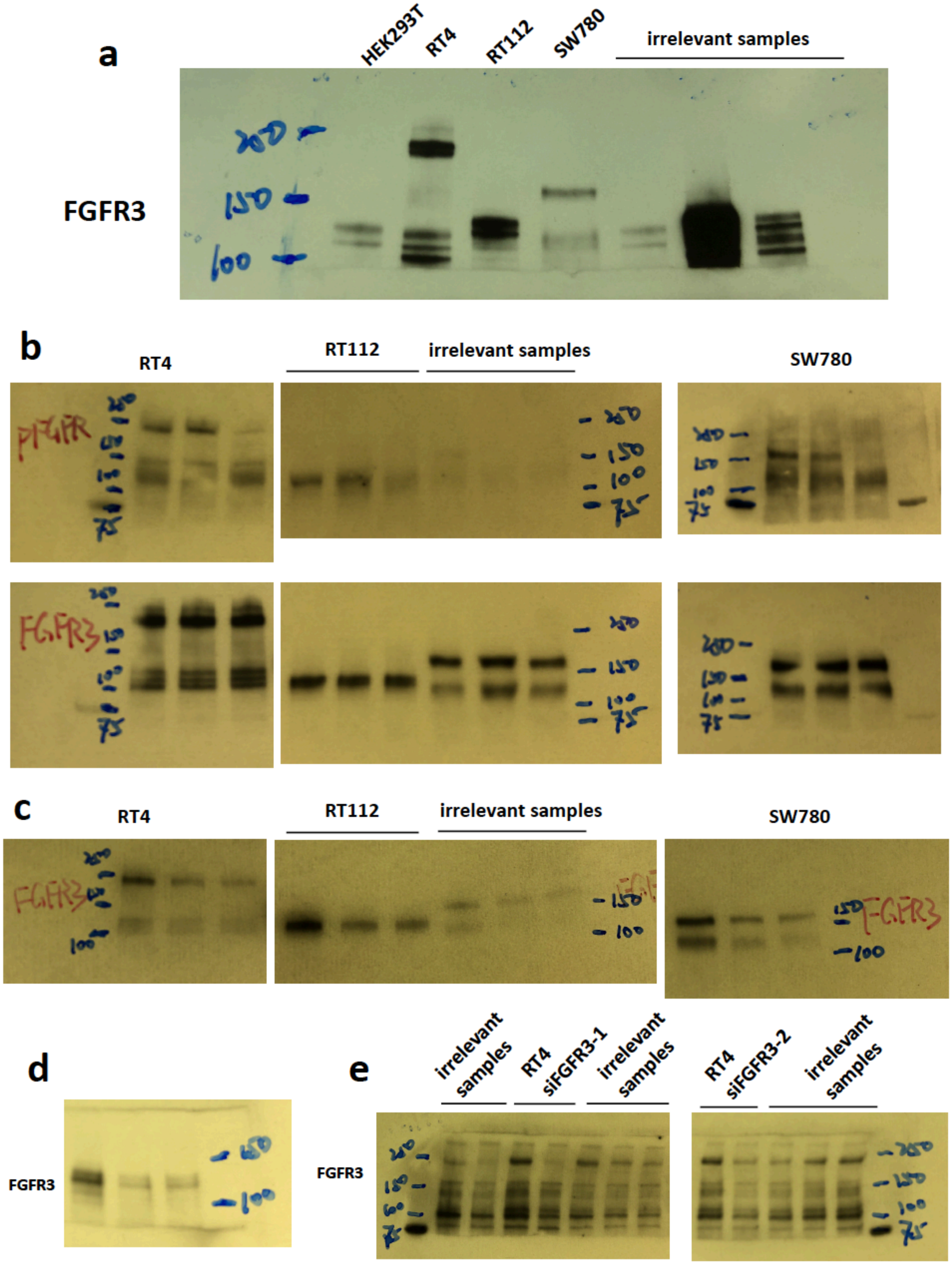
a
HEK293T
RT4
RT112
SW780
irrelevant samples
FGFR3
b
RT4
RT112
irrelevant samples
SW780
c
RT4
RT112
irrelevant samples
SW780
d
FGFR3
e
irrelevant samples
RT4 siFGFR3-1
irrelevant samples
RT4 siFGFR3-2
irrelevant samples
FGFR3

f

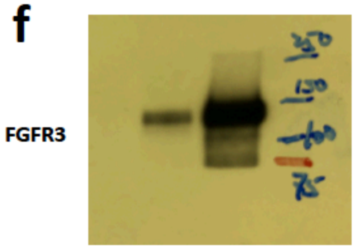
FGFR3

g

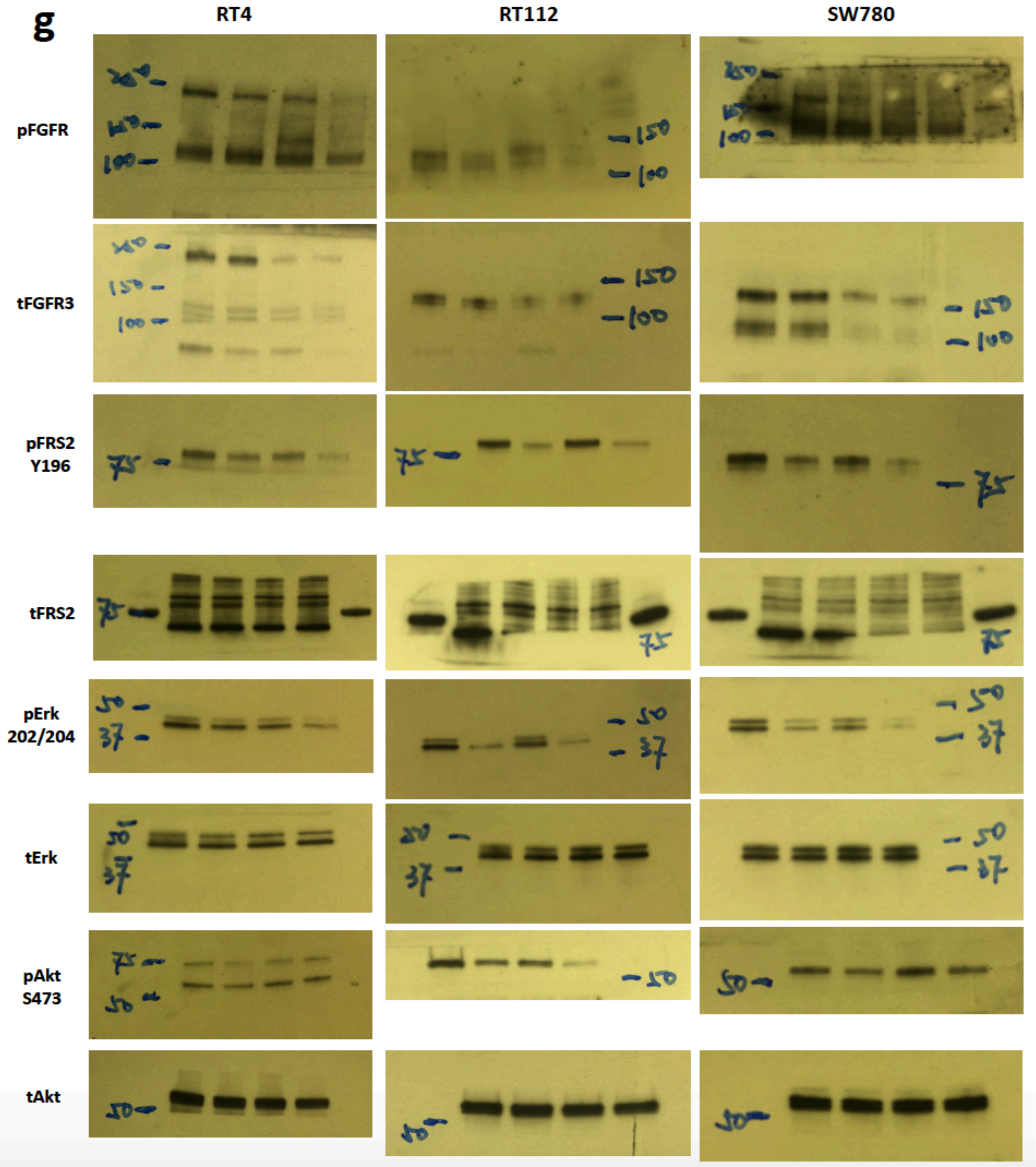
RT4
RT112
SW780
pFGFR
tFGFR3
pFRS2
Y196
tFRS2
pErk
202/204
tErk
pAkt
S473
tAkt

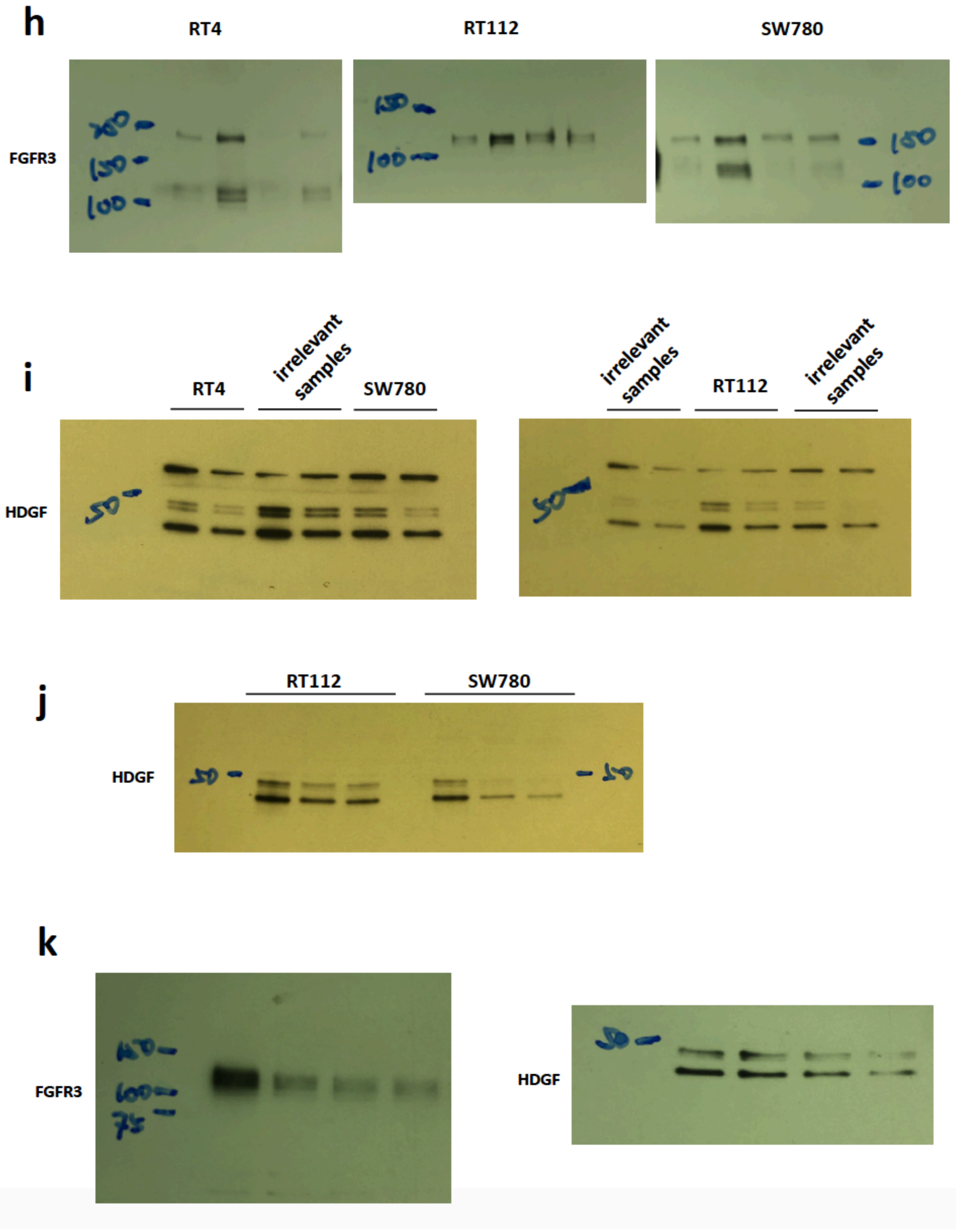


**Supplementary Figure 13. Uncropped western blots.**

**a,** Uncropped western blots for main figure 1b. **b,** Uncropped western blots for main figure 3a. **c,** Uncropped western blots for main figure 3b. **d,** Uncropped western blots for main figure 3c. **e,** Uncropped western blots for main figure 3e. **f,** Uncropped western blots for main figure 3g. **g,** Uncropped western blots for main figure 4. **h,** Uncropped western blots for main figure 5g. **i,** Uncropped western blots for main figure 6c. **j,** Uncropped western blots for main figure 6f. **k,** Uncropped western blots for main figure 7d.

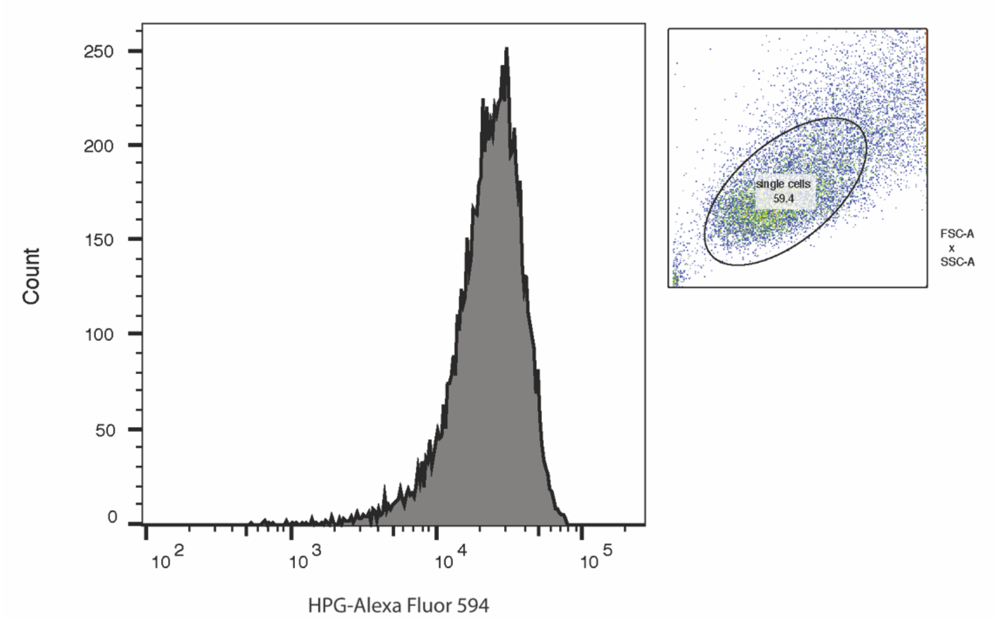


**Supplementary Figure 14. Flow cytometry gating strategies for total protein translation.** Flow cytometry gating strategy of HPG-Alexa Fluor 594 for main figure 5e. Results were first gated on FSC-SSC for single cells. And then Alexa Fluor 594 signal of the single cell population was further analyzed.
